# Closing the Loop for Epoxy Composites: Sustainable Routes for Chemical Recycling of Anhydride-Cured CFRPs

**DOI:** 10.3390/polym18202583

**Published:** 2026-10-20

**Authors:** Ehsan Zolfaghari, Fabian Rechsteiner, Emanuela Muscolino, Nadka Tz. Dintcheva, Clelia Dispenza

**Affiliations:** 1 Dipartimento di Ingegneria, Università degli Studi di Palermo, Viale delle Scienze, ed. 6, 90128 Palermo, Italy; ehsan.zolfaghari@unipa.it (E.Z.); emanuela.muscolino@unipa.it (E.M.); nadka.dintcheva@unipa.it (N.T.D.); 2 Fraunhofer Institute for Casting, Composite and Processing Technology, IGCV, Am Technologiezentrum 2, 86159 Augsburg, Germany; fabian.rechsteiner@igcv.fraunhofer.de

**Keywords:** carbon fiber-reinforced plastics (CFRPs), anhydride-cured epoxy resin, chemical recycling, green solvents

## Abstract

Carbon fiber-reinforced plastics (CFRPs) combine outstanding strength-to-weight and stiffness-to-weight ratios with long-term durability, enabling significant weight reduction, energy savings, and emission mitigation compared with conventional structural materials. Their rapidly increasing use in aerospace, mobility, infrastructure, and energy applications, however, raises critical challenges for end-of-life management. While mechanical, thermal, and chemical recycling routes for CFRPs have been widely investigated, comparative assessments often emphasize degradation efficiency or fiber recovery while giving limited attention to matrix chemistry and safety, health, and environmental (SHE) implications. This review focuses on the chemical recycling of anhydride-cured epoxy resin systems, which dominate many high-growth CFRP applications. The various solvolysis and depolymerization strategies are critically examined in terms of reaction conditions, targeted bond-cleavage pathways, and the chemical structure and reusability of the resulting degradation products. Unlike previous reviews, this work integrates mechanistic analysis of anhydride-cured epoxy degradation with a quantitative evaluation of the SHE profiles of solvents and co-reactants, enabling comparison of chemical recycling routes beyond process efficiency alone. By jointly addressing chemical performance, circularity potential, and safety considerations, this review provides a framework for assessing and guiding the development of more sustainable chemical recycling strategies for CFRP systems.

## 1. Introduction

Carbon fiber-reinforced plastics (CFRPs) are metamaterials offering some of the highest strength-to-weight and stiffness-to-weight ratios of all structural materials. Important weight reductions, and hence energy savings and lower emissions, result from replacing traditional structural materials such as aluminum and steel with CFRPs. CFRPs also have high Young’s modulus, dimensional stability and superior chemical and corrosion resistance. Over the past few decades, they have become key strategic materials in many high-tech industries, including aerospace, military, electronics, marine industry, sports equipment, and renewable energy. They are now also being increasingly used in light-duty trucks and electric cars [1,2,3,4,5,6,7].

Although CFRPs offer significant advantages over more traditional materials, they also present certain challenges. Their manufacturing process is very energy-intensive, mainly due to the high temperatures required to produce carbon fibers (up to 1600 °C for the standard fibers, even higher for the high modulus ones) [8]. In the transportation sector, for example, the net energy impact of these materials depends on the initial energy cost, which can be very high, and on the fuel savings that accumulate over time during product use. It may take many years of product use for the accumulated fuel savings to fully offset the production energy. In some cases, energy recovery can never be complete [9].

Other issues are related to the lack of convenient end-of-life (EOL) solutions due to the difficulty of recycling, particularly CFRPs with thermoset matrices [4,5,6,7]. EOL solutions for composite products that enable fiber reclamation could reduce the energy costs associated with manufacturing—if not for the same composite materials, then for other fiber-reinforced composites, with less demanding performance requirements. However, the energy trade-off is complicated by the fact that there are as many CFRPs as there are performance requirements and manufacturing technologies because these factors influence the choice of matrix, fiber fraction, fiber format and orientation, temperatures and cure times, as well as the incidence of scrap on production volumes. Therefore, these factors also affect production costs and the attractiveness of the various EOL solutions. In addition to these considerations, environmental taxes, incentives and regulations aiming to reduce global anthropogenic greenhouse gas emissions to ‘net zero’ by 2050 must be taken into account as strong drivers for identifying techno-commercial solutions for the CFRP industry [10,11,12].

In recent years, numerous original and review papers have been published on various approaches to recycling carbon fiber-reinforced composites. These papers can help inform the choice between the three main options—landfill disposal, incineration with energy recovery, and recycling—based on their impact on climate change, resource consumption, environmental quality, and human health [13,14,15,16,17] (Figure 1). In most impact categories, incineration is not much less burdensome than landfill. In particular, the environmental benefits depend on the quality of the emissions produced by the incinerators employed. Moreover, the benefits of energy recovery can be significant or minimal, depending on a region’s primary energy source. Using recovered energy can only be advantageous in terms of avoiding CO_2_ emissions when it is produced mainly from fossil fuels and not from renewable sources or nuclear power plants. Conversely, the environmental impact of landfill is based on the assumption that these materials are not easily degraded [18,19,20]. Although recycling is an attractive approach, it is fragmented into many different methodologies that are developed and tested only on specific material systems. These methodologies are not always described or characterized with sufficient accuracy and detail to determine their general validity. Their attractiveness can be significantly affected by the source of composite waste and their volumes. Moreover, the impacts of recycling are strongly linked to the selected recovery process and secondary applications in which the recovered materials are used.

After a very short presentation of the structural properties of carbon fibers, this review focuses on the chemical recycling of anhydride-cured epoxy resin systems, which are among the most widely used matrices in CFRPs for mobility, infrastructure, and energy applications. Unlike many existing reviews that treat epoxy resins as a single, homogeneous class or primarily emphasize fiber recovery efficiency, this work adopts a matrix-centric perspective. Specifically, it links curing chemistry and network structure to degradation pathways, bond-selective cleavage, and the chemical nature and reusability of the resulting products. In addition, this review uniquely integrates quantitative safety, health, and environmental (SHE) assessments of solvents and co-reactants with mechanistic and process-level considerations. By combining degradation chemistry, circularity potential of recovered matrix products, and SHE performance within a unified framework, this review aims to support informed comparison of solvolysis strategies and to guide the development of safer and more sustainable chemical recycling routes for anhydride-cured CFRP systems.

### 1.1. Carbon Fibers

Carbon fibers (CFs) are among the lightest inorganic materials available on Earth, resulting from strong covalent bonding between sp2 hybridized carbon atoms. They are not homogeneous anisotropic materials but rather a collection of anisotropic nanoscale units. At the atomic level, they are formed by imperfect graphene sheets due to vacancies and impurities, with a turbostratic structure. At the mesoscale, they have a polycrystalline structure, with an axial preferred orientation of the Basic Structural Units (BSUs) that can split, twist, fold and assemble with other BSUs. At the micro-scale they have a skin-core structure, with pores and surface texture, often covered by a polymer coating (sizing) [21] (Figure 2).

There are different methods of producing CFs: from fossil-derived feedstocks, like polyacrylonitrile (PAN) and petroleum pitch, and from biomass-derived feedstocks, like viscose and lignin. Pitch- and biomass-based CFs only play a minor role on the global market [21,22], and their processing paths are technologically similar to those of PAN-based CFs [23]. The PAN-based process is the most widely used method, and the market share of PAN-based carbon fiber accounts for about 96–98% [24]. Considering that the global carbon fiber composites market is projected to grow from USD 25.6 billion in 2025 to USD 72.3 billion by 2035, registering a compound annual growth rate (CAGR) of 10.9%, CF demand is also expected to increase [25].

Carbon fibers derived from PAN are manufactured through a multi-step process that begins with the chemical polymerization of PAN, followed by fiber spinning—typically via wet-solution spinning—and a final finishing stage in which surface treatments and sizing are applied to protect the fibers and enhance adhesion with the polymer matrix.

During spinning and subsequent thermal treatments, the precursor undergoes several chemical transformations: dehydrogenation and cyclization at 200–300 °C, carbonization at 1200–1600 °C, and—for high-modulus grades—graphitization at temperatures up to 2500 °C. These steps determine the final carbon content, the orientation of graphite layers, crystallinity, and other microstructural properties of the fibers. The overall process yield is typically 40–50%, and the main gaseous emissions include hydrogen cyanide (HCN), carbon monoxide (CO), and ammonia (NH_3_). The energy consumption of carbon fiber production is in the range 198–1132 MJ/kg [8,26]. The substantial variability arises from several sources, including differences in precursor type, production technologies, and the methodological choices made in life-cycle assessments. A detailed cost and energy breakdown for each stage of production is provided in the recent critical review and meta-analysis by Chen et al., which evaluates the energy demand, carbon footprint, and broader environmental impacts of carbon-fiber manufacturing [8].

### 1.2. Epoxy Resins

The choice of matrix can significantly influence the performance characteristics of the final composite (e.g., strength, stiffness, hardness, heat resistance, chemical and solvent resistance) but also the choice of the manufacturing process, the efficiency of recycling, and the quality of the reclaimed carbon fiber. Common matrices used with CF to produce CFRPs include thermoset polymers, such as epoxy resins, polyester resins, vinyl ester resins, and thermoplastics like polyetheretherketone (PEEK), polyetherimide (PEI), polyetherketoneketone (PEKK), polypropylene (PP) and polyamide-6 (PA6). Choosing between thermoset and thermoplastic resins depends on the specific requirements of the application, including mechanical and thermal properties, production volumes, cost, and environmental considerations [27,28,29,30,31,32,33].

Epoxy resins are common matrix materials used in CFRPs for their excellent thermo-mechanical properties and strong adhesion to CFs. The final properties of cured epoxy resins are determined by the choice of epoxy monomers and curing agents (also named hardeners), as well as by the specific curing conditions, which drive the reactions to yield a rigid, three-dimensional crosslinked network [28,34].

The most common type of epoxy monomers used in industry is based on diglycidyl ether of bisphenol-A (DGEBA) and its oligomers (see Figure 3a), produced by DGEBA homopolymerization in the presence of suitable catalysts [35]. They can be either liquid or solid, depending on their molecular weight, and require a polyfunctional hardener to form a network. The commercial epoxy resins are often mixtures of different oligomers. They can also contain impurities that derive from the production process (polyols, chlorine derivatives, branched monomers). Glycidyl ether epoxy monomers can also be derived from other phenols, such as bisphenol F (Figure 3b), resorcinol, brominated and fluorinated bisphenols, and phenol–formaldehyde novolacs (the term coming from Swedish “lack” for lacquer and Latin “novo” for new, because they were initially envisioned as a replacement for natural lacquers) (Figure 3f). Multifunctional epoxy monomers are widely used in high-performance composites because they tend to increase the cross-linking density. For instance, N,N,N’,N’-tetraglycidyl-4,4′-methylenedianiline (TGDDM) (Figure 3c) is a typical monomer extensively used in aerospace composites. Epoxy-functionalized phenolic polymers are also common and are known to yield epoxy resins with a higher glass transition temperature (Tg) and high resistance to thermal degradation [36]. Cycloaliphatic resins (Figure 3d,e), another class of epoxy resins, have less tendency toward yellowing than aromatic resins. Therefore, they are more often used in combination with glass fibers, for aesthetic reasons. For their low viscosity and electrical loss properties, they are also widely used as encapsulants in electronics [35,37].

In order to cure these resins, a variety of curing agents are used that can react either with the oxygen atom (electrophilic attack) or the methylene group (nucleophilic attack) of the epoxy monomer. Hardeners have reactive groups like carboxylic acids or hydrogen atoms bound to nitrogen. Anhydrides (Figure 4(a1–a7)), amines (Figure 4(b1–b5)), and polyamides are the most common hardeners [38]. The choice of the hardener has a major impact on curing time and temperature, as well as on the final properties of the products [35]. The anhydrides employed range from dicarboxylic acid cyclic anhydrides to tetracarboxylic acid cyclic dianhydrides, with the latter yielding higher cross-link density and, consequently, enhanced thermal, chemical and solvent resistance. Linear aliphatic polyanhydrides are used as flexibilizing modifiers. Accelerators are chemicals such as tertiary amines (e.g., 2,4,6-tris(dimethylaminomethyl)phenol (DMP-30), benzyldimethylamine (BDMA)) and triethylamine (Et_3_N) in Figure 5(a1–a3), and imidazoles (e.g., 2-methylimidazole (2-MI)) in Figure 5b, which accelerate the epoxy–anhydride reaction. These catalysts initiate the epoxy–anhydride reaction by promoting anhydride ring-opening, thereby reducing the reaction temperature and time while preserving high cross-link density and final properties.

Epoxy–anhydride systems are widely used due to their outstanding performance. When anhydrides are employed as curing agents, the resulting epoxy composites exhibit high chemical and thermal resistance, elevated Tg, and excellent overall durability. These matrices also offer long pot life, low toxicity, and rapid cure kinetics. Additional advantages include low curing shrinkage, limited heat generation during the curing reaction, and high dielectric strength, all of which contribute to their broad applicability [35,39,40,41]. All these features make epoxy–anhydride systems particularly attractive for producing strong yet lightweight composite materials used in construction and aerospace. In the wind energy sector, anhydride hardeners have also been employed in the manufacture of wind turbine blades [35].

### 1.3. Epoxy–Anhydride Curing Reactions

Common epoxy monomers that react with anhydride curing agents include bisphenol-A epoxy resins, bisphenol-F epoxy resins, novolac epoxy resins, cycloaliphatic epoxy resins, and glycidyl amine epoxy resins. Among the earliest hardeners used with epoxy resins were dicarboxylic acid anhydrides, with cycloaliphatic anhydrides representing the most important class within this group.

The epoxy–anhydride curing process is quite complex due to several possible competing reactions. In Figure 6, the main reactive steps, when no accelerators are used (mechanism A) and when a Lewis base is used as an accelerator (mechanism B), are shown. In mechanism A, the first step is the anhydride ring opening by a hydroxyl group of the epoxy backbone. Then, the newly formed carboxyl group of the anhydride reacts with an epoxy group of the resin, forming a hydroxyl diester. The hydroxyl diester reacts with the anhydride to generate another carboxyl group, or with an epoxy monomer forming an ether bond. This last reaction occurs especially at higher temperatures. The ratio between ester bonds and ether bonds can also be controlled by the stoichiometry of the two monomers. Without accelerators, the curing reactions are slow and often incomplete. In addition, some side reactions such as esterification between hydroxyl and carboxylic acid groups that release water molecules, and epoxy hydrolysis in the presence of moisture, can occur [35,42].

In the presence of a Lewis base accelerator (mechanism B), the formation of carboxyl anions from anhydride is catalyzed by nucleophilic activation of the anhydride. The carboxylate anion reacts with the epoxy monomer forming an alkoxide ion that ring-opens an anhydride group to generate a carboxylate anion again. Continuation of these alternating steps results in a polyester. Etherification between epoxies and alkoxide anions is less likely and occurs at higher temperatures [35,42].

When using imidazoles as catalysts (mechanism C), they form imidazolium–alkoxide ion pairs by opening epoxides. The alkoxide reacts with the anhydride and propagates the esterification, while the active ionic species is regenerated. The reactions are epoxide-activated and occur at lower temperatures.

Lewis acids can also be used as catalysts. The curing reactions do not proceed via the alkoxide intermediate but by electrophilic activation of both the epoxy ring, making ring opening easier, and the anhydride carbonyl group, making it more susceptible to nucleophilic attack. The reactions are more controlled than when Lewis bases are used [35].

### 1.4. CFRP Manufacturing Processes

The intended application of a composite material defines the requirements in terms of fiber fraction, fiber orientation and choice of resin and, to a large extent, the manufacturing process. Long fibers and higher fiber fractions are generally used for high-performance structural components, whereas short fibers and lower fiber fractions can be used in non-structural or semi-structural parts. The production method is also determined by the component’s size, shape, and finish requirements.

Among all industrial composite manufacturing processes, pultrusion, filament winding, resin transfer molding (RTM), and vacuum assisted resin transfer molding (VARTM) are mainly used with thermoset polymers and long fibers. On the other hand, compression molding (with both short or long fibers), injection molding with short fibers and 3D printed continuous-fiber composites typically use thermoplastic matrices. Autoclave molding of pre-impregnated fabric laminates is the preferred process for high-performance aeronautic and aerospace applications [28,43,44,45,46].

Energy demand for thermoset CFRP manufacture varies markedly across the various processes. Continuous processes such as pultrusion and filament winding exhibit the lowest energy intensities (≈2–4 MJ/kg) due to localized heating and minimal thermal losses. Closed-mold processes—including RTM, VARTM, and cold-press molding—occupy a middle range (≈8–12 MJ/kg), benefiting from efficient heat transfer and moderate cure temperatures. The highest energy demands are associated with prepreg curing, particularly when autoclave processing is used; combined with the intrinsically energy-intensive production of prepregs, total process energy can exceed 60–70 MJ/kg [47]. The typical fiber fractions, consolidation (porosity) degree and temperature of cure of different manufacturing processes affect the composite material glass transition temperature, and consequently the ease of matrix degradation, as will be discussed in the following section.

## 2. Overview of CFRP Recycling Methodologies

Recycling of epoxy resin CFRPs has been challenging due to their strong chemical bonds and diverse chemical structures. The recycling methods can be classified as “mechanical”, “thermal” or “chemical” (Figure 7), depending on whether the main approach is mechanical, such as in cutting and sectioning, shredding, crushing, and milling; thermal, using heat, generally after a mechanical pre-treatment; or chemical, using solvents, chemical co-reactants and/or catalysts, generally in combination with heat and following a mechanical pre-treatment.

### 2.1. Mechanical Recycling

The mechanical recycling approach, employing a hammer mill or similar equipment, reduces waste composite materials to smaller and more uniform pieces. The crushed composite can find application in reinforced plastic formulations, thus reducing the need for virgin raw materials and increasing resource efficiency [13,16,47,48,49]. For instance, Song et al. utilized mechanically recycled CFRPs to reinforce epoxy foams [50]. The characterization of these secondary composites showed a notable enhancement (~85%) in the mechanical properties with respect to neat epoxy foam. In terms of energy consumption, mechanical recycling has the lowest energy consumption among all recycling routes (0.27 to 2.03 MJ/kg) [47]. The main disadvantage is that crushing and grinding the composite can damage the fibers, causing internal fracture and delamination, deteriorating their mechanical properties, and can also damage the matrix–resin interface, causing the resin to detach. The material obtained is then very heterogeneous in terms of particle size, aspect ratio, composition, and resulting properties.

The deterioration of material properties restricts the recyclate to lower-performance, lower-value applications, limiting the overall value recovery of the process. Moreover, the tools required to cut these materials typically rely on costly diamond blades, meaning that, even if energy consumption is modest, the capital and maintenance costs of the equipment are not negligible [16,28,49].

Often, mechanical grinding is the first step before thermal or chemical recycling steps that have the function of digesting the epoxy resin matrix. In this case, both the energy consumption and the overall processing impacts and costs add to the costs and impacts of the following step.

### 2.2. Thermal Recycling

Thermal recycling methods include pyrolysis [51,52,53], thermal oxidative degradation [54] and fluidized-bed approaches [55,56,57]. These approaches induce the degradation of resin at high temperatures. They allow the recovery of “clean” carbon fibers with longer lengths, which have a higher quality compared to the short and uneven size fibers mixed with resin obtained by mechanical recycling. The fiber length is mainly controlled by the cutting of the composite parts before the thermal treatment and their orientation within the part.

In pyrolysis, CFRP undergoes high-temperature treatments in a (nearly) oxygen-free environment (N_2_ or Ar atmosphere). Typical temperatures range from 450 to 700 °C, where the resin matrix breaks down into gases, oils and char. By recovering the energy from the incineration of the resin matrix, the energy demand for the process itself can be partially compensated [51,52,53].

While pyrolysis degrades the resin by thermal cracking, fluidized bed techniques use oxidative degradation, exposing the cut or shredded CFRPs to a hot stream of air or sand fluidized by air. The latter process is more efficient in yielding clean fibers, with no char formation on the fiber surface. In contrast, it requires fine shredding of the composite pieces (size < 25–50 mm), consumes more energy and induces more damage to the fibers (the fiber strength retention is 60–80%, compared to the 85–95% of pyrolysis) [55,56,57].

The resulting pyrolysis gases can be condensed into a mostly aromatic oil. The composition depends on the specific polymer type, but it is mostly made up of carbon, hydrogen and nitrogen with a calorific value around 37–39 MJ/kg. The oil can be used as a chemical feedstock material.

All thermal recycling methods are largely insensitive to matrix type and can be effectively applied to a wide range of resins [51,52,53,54,55,56,57]. Their main drawbacks include the high energy demand required to reach the operating temperatures (≈30 MJ/kg) [13], and lower quality of reclaimed fibers, if process conditions are not properly controlled [13,51], as well as the comparatively high capital cost of the equipment and additional expenses associated with treating the toxic and greenhouse gases generated during resin decomposition [13,51,52,53,54,55,56,57].

In recent years, molten salt and molten alkaline recycling methods have been investigated to study the application of eutectic systems such as NaOH–Na_2_CO_3_, NaOH–KOH, ZnCl_2_, and carbonate mixtures (Na_2_CO_3_–K_2_CO_3_–Li_2_CO_3_) as reaction media to degrade epoxy composites at 285–450 °C [58,59,60,61,62,63,64]. Compared to pyrolysis (450–700 °C), these approaches operate at lower temperatures while showing high resin removal efficiencies (>97%) and retaining carbon fiber tensile strength (up to 95–99%) [58,59,60,61,62,63,64]. For instance, Nie et al. employed molten KOH treatment at 285–330 °C and enabled complete resin decomposition with >95% fiber strength retention [63], while carbonate-based molten systems (Shu et al.) [58] and NaOH–Na_2_CO_3_ mixtures (Ren et al.) [61] achieved comparable resin removal (>97%) and strength retention approaching 99% at ~350–400 °C [58,59,60,61]. The enhanced performance is attributed to improved heat and mass transfer in the ionic melt and catalytic cleavage of C–N and C–O bonds within the crosslinked epoxy network.

The energy demand is generally lower than in conventional pyrolysis (~30 MJ/kg), because the degradation proceeds through a chemically assisted approach rather than purely thermal cracking [60,61,62]. In addition, contamination of the composite, such as paint and attached thermoplastics, does not have a major effect on the efficiency of molten salt systems [58,59,60,61,62]. On the other hand, some issues include the need for salt recovery and regeneration, potential equipment corrosion, and surface oxidation of recovered fibers [63]. Several molten salt processes reported the generation of by-products such as H_2_ and CO at elevated temperatures or liquid-phase products including phenols and other aromatic compounds after epoxy depolymerization [58,59,60,61,62,63].

Recovered fibers have been employed in high-performance thermoplastics such as polyether ether ketone (PEEK), resulting in composites with high tensile strength, flexural strength, and interlaminar shear strength comparable to those reinforced with virgin fibers [59,60,61].

Overall, molten salt and molten alkaline processes provide efficient matrix depolymerization at moderate temperature ranges (285–450 °C) and high fiber quality retention (95–99%) compared to pyrolysis.

### 2.3. Chemical Recycling

The term chemical recycling encompasses all technologies that break down the chemical bonds of a polymer matrix through chemical reactions. Under this definition, pyrolysis and fluidized-bed treatments could also be included, as they induce polymer depolymerization and gasification. However, these technologies are traditionally classified as thermal methods because heat provides the dominant energy input. Furthermore, other substances—such as solvents, co-reactants and catalysts—are not used, and the degradation products are not typically recovered for reintegration into manufacturing processes [63,65,66].

In chemical recycling, the main driving force is instead provided by the chemical energy of solvents and co-reactants, which are often recovered and reused together with the monomers and oligomers generated during resin digestion [41,67]. Thermal activation is frequently also required, but the defining feature of these processes is the central role of the solvent-mediated reactions and the opportunity for the recovery and reuse of resin in the supply chain [41,67,68].

Chemical recycling methods can be broadly classified into harsh and mild approaches. Harsh chemical recycling operates under severe conditions—such as high temperatures and elevated pressures—which promote extensive chain scission and allow the breakdown of highly crosslinked matrices [63,65,66,69]. In contrast, mild chemical recycling usually relies on the solvating power of a liquid, often combined with co-reactants and catalysts, to compensate for the lower energy supply and/or longer processing times to degrade any resin present and expose the fibers for reclamation [39,41,67,70]. There is an inherent need for solvolysis to be specific to the type of waste being fed into the process and to address the cleavage of specific chemical bonds and the formation of the most appropriate monomers and oligomers [41,67,68]. In the context of epoxy/anhydride resins, the bonds to be cleaved are essentially ester and ether bonds [39,41,68], but the various approaches can lead to different degradation products, which can be easier or more difficult to separate from solvents and co-reactants [41,67,68]. Moreover, these products may or may not be reintegrated in the resin mixture.

Depolymerization of epoxy–anhydride resins primarily targets the ester linkages formed by the ring opening of cyclic anhydrides by epoxy groups and the ether bonds arising from epoxy monomer homopolymerization and other side reactions [35,68,71]. Among these, ester bonds are the most susceptible to cleavage [66,70,72]. Different types of chemical reactions can be promoted to cleave these bonds, including hydrolysis [72,73,74], alcoholysis [75,76,77], glycolysis [78,79], aminolysis [66,67,70] and acetolysis [68] (Figure 8). Ether bonds exhibit higher bond dissociation energies and are therefore more difficult to activate; their cleavage typically requires concentrated strong acids, extended residence times, and often pressurized reactors, or metal catalyst-supported reductive pathways [68,80].

In the following sub-sections, examples of the different approaches are presented, with information provided on (i) the nature of the epoxy monomer and anhydride; (ii) the Tg of the resin or composite as an indicator of the resin/matrix crosslinking degree and density; (iii) the solvent(s) employed and any catalysts used for the chemical recycling approach followed; (iv) the optimal reaction conditions and the corresponding degree of resin degradation; (v) the quality of the reclaimed fibers, where applicable; (vi) evidence of a possible reuse of the degraded matrix products; and (vii) the proposed degradation reaction mechanism, when described. The most relevant conditions and main findings are summarized in Table 1, Table 2, Table 3 and Table 4.

#### 2.3.1. Hydrolysis

In hydrolysis reactions, the nucleophilic attack by water on the ester bonds yields degradation products enriched in hydroxyl and carboxyl groups at relatively high temperatures and pressures [73,81,82]. Such reactions may proceed under milder conditions when nucleophiles stronger than water (such as hydroxide ions) are employed. In alkaline media, due to the presence of metal hydroxides, saponification occurs, leading to carboxylate salts that may precipitate [73]. By contrast, the use of supercritical water offers the advantage of reduced polarity, which enhances matrix swelling and the solubility of the degraded products, but also creates a highly oxidizing and corrosive environment that can be harsh on the fibers and the reactor [81,82].

The earliest studies in this field refer to epoxy–anhydride degradation as an undesired phenomenon in humid/aqueous environments, as early as the 1970s–1990s. The first report on the use of water to decompose epoxy resins was published by Fromonteil et al. in 2000 [83]. The resin was a diglycidyl ether of bisphenol-A/polyamine–fatty polyamide mixture and water was used under both sub- and supercritical conditions. This is not the type of resin system this review focuses on, and it is reported here because it represents the first documented example of epoxy-network hydrolysis. No significant degradation was observed at temperatures below 200 °C. At temperatures between 300 °C and 400 °C, the formation of low-molecular-weight compounds in the liquid phase was detected. Complete digestion of the epoxy resin was achieved at 410 °C and 24 MPa within 60 min. The digestion process produced species that were either dissolved or suspended in the aqueous phase, together with gaseous end products.

The earliest explicit report on the hydrolytic degradation of an epoxy–anhydride resin using near-critical water is by Liu et al. [81], who investigated the decomposition kinetics of a DGEBA epoxy monomer cured with MHHPA in near-critical water at 260–300 °C under pressures of approximately 3.0–6.8 MPa, using 1 g of epoxy resin in 5 mL of water in a stainless-steel batch reactor without additives. This study reported a complete degradation within 30 min at temperatures ≥280 °C. Later, Gong et al. [82] examined the degradation products of a similar resin degraded under the same conditions (1 g epoxy/5 mL water, 260–300 °C) and identified phenolic and bisphenol-A derivatives via gas chromatography–mass spectrometry (GC–MS), proposing cleavage of both ether and ester bonds.

In 2014, Liu et al. [73] documented an acid-catalyzed and an alkali-catalyzed degradation process for a similar epoxy resin system (with H_2_SO_4_ or KOH, respectively) in near-critical water (270 °C) for 45 min (1 g of resin in 10 mL of water), achieving 97.7–100% decomposition. They concluded that KOH favored ether bond cleavage, while H_2_SO_4_ promoted both ether and ester hydrolysis.

When using a phosphotungstic acid (HPW), a very strong Brønsted acid, a DGEBA epoxy monomer cured with nadic methyl anhydride (NMA) system (Tg = 168.4 °C) was degraded in a 100 mL Parr pressure reactor at 160–200 °C (Liu et al.) [72]. The optimal conditions were 190 °C for 5 h with 1 wt% HPW (0.2 g HPW in 19.8 g water) and 2 g cured epoxy in a 100 mL Parr reactor. Temperatures below 180 °C did not allow for sufficient solvent diffusion to take effect within the selected reaction time, whereas at 190 °C the resin entered a rubbery state that facilitated chain scission. Complete degradation was achieved with 1 wt% HPW, yielding yellow, organo-soluble degraded matrix products (DMPs) (in acetone or tetrahydrofuran). Nuclear magnetic resonance (NMR), Fourier transform infrared spectroscopy (FTIR), and elemental analyses confirmed selective cleavage of ester bonds and formation of hydroxyl- and carboxyl-containing species. The blend of DMPs with fresh DGEBA at 5–50 wt% was cured with nadic methyl anhydride (NMA) as hardener (epoxy/anhydride molar ratio equal to 1:0.8), and 2-ethyl-4-methylimidazole as catalyst (1 wt% with respect to NMA). The Tg of the cured resin with 5 wt% DMPs increased to Tg = 177.4 °C, then decreased to 113.5 °C with a further increase in DMPs up to 50 wt%. Dynamic mechanical analysis (DMA) showed that the rubbery plateau modulus increased for 5–10 wt% DMPs and decreased at higher loadings. Flexural and impact strengths improved up to 30 wt% and 10 wt% DMP, respectively, before decreasing. In conclusion, the authors documented that up to 10 wt% DMPs do not substantially compromise either thermal or mechanical performance of the resin [72].

**Table 1:** Hydrolysis routes for epoxy thermoset solvolysis.

Reference	Digested System	Size/ Weight	Tg (°C)	Solvent Catalyst	Optimal Conditions	Degrad. (%)	Cleaved Bonds	Restored Functionality	Recyclable DMP	Recovered CF Quality
Liu et al., 2012 [81]; Gong et al., 2013 [82]	DGEBA-MHHPA neat resin	1 g	54.3	Near-critical water	280–300 °C, 30 min	100	Ester, ether	OH (phenolic)	n/r	n/a
Liu et al., 2014 [73]	DGEBA-MTHPA neat resin	1 g	n/r	Near-critical water/KOH or H_2_SO_4_	270 °C, 45 min	97.7–100	Ester, ether	OH, COOH	n/r	n/a
Liu et al., 2017 [72]	DGEBA-NMA neat resin	2 g	168.4	Water/phosphotungstic acid	190 °C, 5 h, 1 wt% HPW	≈100	Ester	OH, COOH	Up to 40 wt%	n/a
Kim et al., 2019 [74]	CFRP Prepregs	1 cm^3^	n/r	Supercritical water	405 ± 2 °C, 120 min, 28 ± 1 MPa	99.5	Ester	OH (phenolic)	n/r	Clean surface; Tensile strength ↓ 18–36%; Young’s modulus ↓ 7.2–20.2%
Backens et al., 2025 [84]	DGEBA-MTHPA neat resin	18.6 g	122.7	Subcritical water	300 °C, 30–45 min	100	Ester	OH, COOH	n/r	n/a

Note: n/r = information not reported; ↓ = decrease in the measured property, by the stated percentage.

Kim et al. [74] investigated the use of supercritical water without added catalysts or oxidants to recycle carbon-fiber-reinforced anhydride-cured epoxy composites. CFRP samples (~1 cm^3^) were treated at various temperatures above the critical point of water (critical temperature of 374 °C and critical pressure of 22.1 MPa). Samples were treated at 405 ± 2 °C and 280 ± 10 bar (28 ± 1 MPa) for reaction times between 10 and 120 min. Owing to its high diffusivity, low viscosity, lower polarity, and rapid heat and mass transfer, SCF-W penetrated efficiently into micro-defects within the CFRP structure, enabling resin swelling and degradation. Scanning electron microscopy (SEM) analysis showed that fibers recovered after 120 min treatment were clean. This result was also confirmed by thermogravimetric analysis (TGA). Raman spectroscopy indicated increased graphitization of the fibers due to the high temperature treatment. Single-fiber testing showed tensile-strength reductions of 18–36% and modulus reductions of 7.2–20.2%, depending on the treatment condition. In contrast to Liu et al. [72], Kim et al. [74] operated at much higher temperatures (405 °C) under fully supercritical conditions without catalysts and focused on approximately complete resin removal and fiber recovery rather than selective bond cleavage or polymer reuse.

In a more recent study, Backens et al. [84] investigated a subcritical hydrolysis of an anhydride-cured epoxy resin (DGEBA cured with tetrahydromethylphthalic anhydride (MTHPA)) (Tg = 122.7 °C) using a high-pressure batch reactor. They systematically studied different temperatures (250–300 °C), times (15–45 min), and water volumes (100–300 mL). Temperature was identified as the dominant parameter, while water volume was statistically insignificant. The study reported that complete decomposition was obtained at 300 °C within 30–45 min. The authors showed that degradation proceeds by heterogeneous surface erosion where polymer bonds degrade faster than water diffusion into the specimen, so the material loss occurred only from the outer surface. This mechanism was validated by dimensional shrinkage and differential scanning calorimetry analysis (DSC). DSC analysis showed that the glass transition temperature at the edges of the specimen (Tg_E_) decreased significantly (87 °C after 15 min at 250 °C), while Tg at the center of the specimen (Tg_C_) remained almost unchanged (121 °C). A core-shrinking model was successfully applied to describe the kinetics, resulting in an activation energy of 80.6 kJ/mol. This study conducted multiple degradation experiments and recorded the mass loss of specimens over time at three temperatures of 250, 275 and 300 °C. By fitting this mass loss data to the core-shrinking model, they determined a specific reaction rate constant for each temperature. Finally, they plotted these rate constants on an Arrhenius graph (lnk’_s_ versus 1/T) and calculated the activation energy from the slope of the resulting linear relationship [84].

#### 2.3.2. Alcoholysis

Alcoholysis involves the reaction of ester linkages with low-molecular-weight alcohols, such as methanol or ethanol, through transesterification mechanisms. In these processes, the alcohol replaces the epoxy-derived alkoxy group, yielding alcohol-terminated epoxy fragments and alkyl esters of the anhydride-derived residues [75,85]. Depending on the reaction conditions, catalysts such as acids, bases or Lewis acids may be employed to enhance the reaction rate. Alcohols are, in general, better nucleophiles than water; hence, they cleave ester linkages faster and at lower temperatures than water, in less corrosive conditions [77,85]. While hydrolysis produces carboxylic acids, which can re-esterify or neutralize alkaline catalysts [73], alcoholysis forms ester-terminated products (transesterification), which are thermodynamically more stable and less prone to recombination, thus favoring a more complete depolymerization [71,76,77]. Some alcohols, e.g., benzyl alcohol, can swell the epoxy network better and penetrate faster than water [71,77]. When comparing this approach to hydrolysis, it must be emphasized that the reliance on organic solvents may raise concerns regarding the electricity and costs of solvent recovery and the overall environmental footprint of the process.

In harsher conditions, alcoholysis can be performed without added catalysts. Okajima et al. [75] reported that supercritical methanol (T > 270 °C, P > 8 MPa) can effectively degrade anhydride-cured epoxy-based CFRP. The study employed both batch-type and semi-flow reactors, processing samples of different sizes (60 × 3 × 3 mm and 200 × 45 × 3 mm). For the batch sample, complete degradation occurred at 270 °C and 8 MPa in 90 min, whereas the larger semi-flow sample was treated at 285 °C and 8 MPa for 80 min, yielding fibers entirely free of residual resin, as confirmed by SEM. Interestingly, matrix-assisted laser desorption ionization–time of flight mass spectrometry (MALDI-TOF/MS) analysis of the degradation products revealed regularly spaced molecular weight distributions, indicating selective ester-bond cleavage and preservation of the main epoxy backbone. The recycled fibers (rCFs) were evaluated using X-ray photoelectron spectroscopy (XPS), tensile testing, and fragmentation testing. Fragmentation tests evaluate fiber–matrix adhesion by measuring the breakage of a single fiber that is fully embedded in fresh matrix. XPS showed reduced hydroxyl groups and increased carbonyl/carboxyl functionalities relative to virgin fibers, implying altered surface chemistry that could affect interfacial bonding. Tensile strength of the rCFs showed only modest reductions (7% for batch-type and 9% for semi-flow reactors), while the fragmentation test showed the interfacial strength of rCFs with fresh epoxy resin decreased by about 20%. The CFRP manufactured using recycled CF and fresh resin exhibited interlaminar shear strength comparable to virgin composites. In contrast, mechanical testing of the recovered resin blended with virgin epoxy resin and cured using HHPA and Et_3_N showed a linear decrease in three-point bending strength with increasing recycled-resin content. Maintaining the recycled resin content in the mixture below 20 wt% allowed the material to retain 80% of its original mechanical performance.

J. Liu et al. [76] explored the degradation of anhydride-cured epoxy resin (Tg = 132 °C) using subcritical methanol with potassium hydroxide (KOH) as a catalyst (alkaline alcoholysis). About 5 g of cured epoxy resin chips (a bar of 80 × 10 × 4 mm cut into chips) decomposed under relatively mild conditions (210 °C, 4.5 MPa, 120 min), with an optimal catalyst concentration of 0.036 mol/L. The degradation products were analyzed using GC–MS, high-performance liquid chromatography (HPLC), electrospray ionization mass spectrometry (ESI-MS), NMR, and MALDI-TOF. GC–MS confirmed the formation of compounds characteristic of transesterification, while HPLC separated three distinct families of degradation products. Based on this comprehensive analysis, the authors proposed that degradation proceeds through three concurrent mechanisms, similar to the one shown in Figure 9: alkali-catalyzed transesterification of ester groups (Path 1), direct base-induced chain scission through reaction with KOH (Path 2), and alkali-catalyzed trans-etherification leading to cleavage of ether linkages (Path 3). The resulting degraded resin fractions (20 wt%) were blended with fresh epoxy resin (DGEBA and MHHPA) and mechanical tests showed that only products derived from an optimal KOH concentration (0.09 mol/L) produced recycled epoxy resins with flexural strength comparable to the virgin resin. Degraded products obtained from all other catalyst concentrations reduced the mechanical performance of the resin, indicating that the reuse potential of degraded resin strongly depends on catalyst loading. Higher KOH concentrations favor the formation and stabilization of carboxylate anions, via path 2 and path 3, that are important reactive intermediates in epoxy–anhydride curing mechanisms, as shown in Section 1.3 and Figure 6.

Ye et al. in 2021 [77] investigated the decomposition of anhydride-cured CFRPs (composite bars of 55 mm × 12 mm × 4 mm, total mass 15 g per batch; 45 wt% resin content in the composite, 120 mL of benzyl alcohol) using a benzyl alcohol/alkaline system. The authors demonstrated that highly efficient degradation (>90%) was achieved at 195 °C, atmospheric pressure and 1:10 weight ratio of NaOH or K_3_PO_4_ to benzyl alcohol, for 40 min of reaction time. Benzyl alcohol acted simultaneously as solvent and co-reactant, promoting transesterification of ester linkages, while NaOH and K_3_PO_4_ supplied a strongly basic environment that further facilitated degradation. The reaction mechanism is shown in Figure 10. K_3_PO_4_ is a weaker base than NaOH and not as strong a nucleophile, but it can still deprotonate benzyl alcohol forming benzylate anions. Benzylate anions readily attack the electrophilic carbonyls in the epoxy–anhydride network, forming a benzyl ester (transesterification) and releasing the corresponding alcohol/acid fragment. After transesterification, the formed ester or the anhydride-derived fragments can undergo saponification with formation of the carboxylate salt and reformation of benzyl alcohol. At 195 °C and in the presence of benzylate anions, ether bonds were also cleaved.

The mechanism is the same when NaOH is used instead of K_3_PO_4_. Both systems preserved high fiber tensile strength [77]. The quality of the recovered carbon fibers was evaluated by SEM, single-fiber tensile testing, and XPS. SEM images showed that fibers retained surface integrity when reclaimed under optimal conditions, while XPS revealed only a slight increase in oxygen content, indicating minimal residual matrix. Single-fiber tensile tests showed modest reductions in strength (approximately 7% with K_3_PO_4_ and 4% with NaOH), demonstrating that the process can recover fibers of high structural quality [77].

The same approach was further refined by Si et al. [71], who optimized the NaOH-to-CFRP mass ratio and achieved near-complete matrix decomposition (98.4%) within one hour at 190 °C and a NaOH:CFRP mass ratio of 1:1. An additional practical advantage of the methodology highlighted in this study is the spontaneous precipitation of the degradation products after 48 h, which enables simple separation and efficient recovery of benzyl alcohol. As in previous studies, the recovered carbon fibers exhibited high quality, retaining approximately 95% of their original tensile strength with only minimal surface oxidation.

Zhao et al. [85] compared the effect of three low-boiling temperature aliphatic alcohols (methanol, ethanol, and propanol) and ethyl acetate, in combination with various alkaline catalysts at a constant resin/catalyst/solvent weight ratio of 1.5:0.5:15 mixed for 3 h with a pulverized MTHPA-cured DGEBA epoxy resin (Tg = 140.8 °C, particle size 0.45–0.9 mm). Methanol (at T > 110 °C) and ethanol (at T > 120 °C) in strongly alkaline media (K_3_PO_4_, K_2_CO_3_, or KOH) achieved degradation degrees above 95%, whereas propanol (T = 110 °C) led to only ~40% degradation. Ethyl acetate was ineffective at 110 °C (~3.5%). In particular, ethanol combined with K_3_PO_4_ at 120–130 °C was identified as the optimal condition, balancing effective resin swelling, transesterification, and catalyst recovery due to the catalyst’s low solubility at room temperature. The alcoholysis products were further hydrolyzed in 1 M HCl at 60 °C, yielding a degraded matrix product (DMP) rich in reactive carboxyl groups that could act as curing agents in new epoxy formulations. Mechanical testing of epoxy resins incorporating hydrolyzed DMP demonstrated properties comparable to the virgin system, even with DMP contents up to ~32%, and glass-transition temperatures remaining above 100 °C at DMP levels approaching 39%. Multi-cycle degradation and recuring tests revealed only a modest performance loss, with tensile strength decreasing by 9.4% after five cycles and Tg stabilizing around 115 °C.

Min et al. [86] reported a recycling approach for anhydride-cured DGEBA/NMA CFRPs (wind blade waste) using a β-phenethyl alcohol 1,5,7-triazabicyclodec-5-ene (TBD) catalytic system under atmospheric pressure. CFRP samples (69 wt% fiber, 20 mm × 10 mm × 5 mm, ~1.6 g) were treated at temperatures between 150 and 200 °C, and the degradation degree showed a clear dependence on temperature. Complete resin removal (100%) was achieved at 190 °C in 2 h and a β-phenethyl alcohol:TBD mass ratio of 30:1 for ~0.25 mol L^−1^ TBD. The study demonstrated that resin removal was a temperature-dependent process, showing less efficiency at lower temperatures. The process efficiency was determined through gravimetric analysis by comparing the mass of the samples before and after the chemical treatment, cleaning, and drying processes. Recovered carbon fibers retained up to 95% of their original tensile strength under optimal conditions, with minimal changes in graphitic structure (confirmed by X-ray diffraction (XRD) and XPS). Ester bond cleavage and formation of aromatic and hydroxyl-containing low-molecular-weight products were supported by FTIR, NMR, and GC–MS analyses. Although the study demonstrated efficient fiber recovery and catalyst/solvent recyclability, closed-loop re-curing of the degraded resin products was not reported [86].

Zhang et al. [87] investigated an alcoholysis process of anhydride-cured epoxy insulating materials (EIM) prepared from DGEBA cured with MTHPA and DMP-30 accelerator. The cured resin was pulverized and reduced to approximately 1 mm particles and degraded in ethanol/K_3_PO_4_ at 120 °C for 3 h under atmospheric pressure, achieving approximately 100% degradation. FTIR analysis confirmed selective cleavage of ester bonds, by reduction of the carbonyl (C=O) peak and the emergence of hydroxyl (–OH) groups, while the ether backbone (–C–O–C–) remained largely intact. As in other studies, the resulting DMP was further hydrolyzed to generate carboxyl (–COOH) groups and then reused to partially replace fresh DGEBA in new epoxy formulations. DMP was used as a partial substitute for fresh DGEBA in a new resin formulation, maintaining the electrical insulation properties when the DMP content was below ~38 wt%. In contrast, thermal properties, tensile strength, and Tg gradually decreased with increasing DMP content. The glass transition temperature (Tg) progressively declined with increasing DMP content, showing a ~39 °C reduction at ~35% content compared to the pure resin. The tensile strength at ~35% DMP content dropped to ~36 MPa, from ~45 MPa for pure resin. The paper reported that tensile strength and Tg were still well retained at 22 wt% DMP.

Compared with earlier benzyl alcohol/NaOH approaches such as that reported by Si et al. [71], a more recent neat-resin study of Liu et al. [88] presents a similar approach to degrade anhydride-cured epoxy resin (DGEBA/MHHPA), only replacing NaOH with K_3_PO_4_. Both strategies rely on selective cleavage of anhydride-derived ester bonds via benzyloxide nucleophiles; this work demonstrated 100% resin degradation under optimal conditions of 190 °C for 60 min. In the CFRP studies, single-fiber tensile tests confirmed that reclaimed fibers maintained about 93–96% of the virgin fiber tensile strength [71,77,86].

The authors also reported reuse of DMP mixed with fresh resin at 10–30 wt%. At 30 wt%, the Tg decreased from ~165 °C to ~103 °C due to reduced crosslink density while at 10 wt%, the Tg reduced to 137.4 °C. Increasing the D-ER content also caused a progressive decline in mechanical properties; at 30% D-ER, the regenerated resin retained a flexural stress of 72.87 MPa. In addition, 10 consecutive degradation–recovery cycles gave average recovery rates of 80.38% for benzyl alcohol and 90.95% for K_3_PO_4_, demonstrating repeated reuse of the recycling system [88].

#### 2.3.3. Glycolysis

Glycolysis is a specific case of alcoholysis in which diols, most commonly ethylene glycol or propylene glycol, act as the nucleophilic agents [78,79,80,81,82,83,84,85,86,87,88,89,90,91,92,93,94]. The ester bonds undergo transesterification with the glycol, producing hydroxyl-terminated epoxy oligomers and glycol-ester derivatives of the anhydride fragments [78,79]. The presence of diols favors the formation of bifunctional products that can be readily reintroduced into new polymer formulations. When alkaline catalysts are added, the increased nucleophilicity of glycolate species significantly accelerates ester cleavage [89,90,91].

One of the earliest scientific reports on transesterification solvolysis is by K. El Gersifi, G. Durand, and G. Tersac in 2006 [92]. They used Ti(IV) n-butoxide (TBT) and diethylene glycol (DEG) as catalyst/solvent to depolymerize DGEBA resin monomer that was cured with hexahydrophthalic anhydride (HP) (Figure 11A) (Tg =132 °C). The depolymerization of both the neat resin and the corresponding CFRP waste proceeded effectively to the monomeric level (see Figure 11B). The glycolysis products consist primarily of ester-diols and tetra-alcohol species, together with residual excess DEG. The polyols were viscous liquids. The excess DEG was readily removed by vacuum distillation without triggering re-equilibration or condensation reactions leading to higher-molar-mass species that were reused for rigid PU foam manufacture [92].

The authors proposed an interesting analogy between the epoxy/anhydride network and polyester thermoplastics, such as polyethylene terephthalate (PET). The latter is formed via polycondensation of terephthalic acid and ethylene glycol. In PET glycolysis, distillation of excess diethylene glycol can induce back-polycondensation, ester-exchange, and cyclization reactions, due to the reversibility of transesterification equilibria, which are catalyzed by residual metal species. These processes ultimately increase the molar mass of the products and reduce monomer purity. In contrast, during epoxy–anhydride glycolysis the degradation products are multifunctional alcohols whose mobility is limited by the presence of aromatic rings and the absence of active catalytic species. Consequently, back-equilibration reactions are kinetically suppressed, allowing excess glycol to be removed by vacuum distillation without the re-formation of higher-molar-mass species [92].

**Table 2:** Alcoholysis routes for epoxy thermoset solvolysis.

Reference	Digested System	Size/ Weight	Tg (°C)	Solvent/ Catalyst	Optimal Conditions	Degrad. (%)	Cleaved Bonds	Restored Functionality	Recyclable DMP	Recovered CF Quality
Okajima et al., 2014 [75]	DGEBA-HHPA CFRP	0.54 cm^3^	n/r	Methanol	270 °C, 8 MPa, 90 min	100	Ester	OH, OCH_3_	<20 wt% reused; ↓ 20% of flexural strength	Clean surface; tensile strength ↓ 7%; interfacial strength ↓ ~20%
Liu et al., 2016 [76]	DGEBA-MHHPA neat resin	5 g	132	Methanol/KOH (0.036 M)	210 °C, 4.5 MPa, 120 min	100	Ester; ether	OH, COOH	20 wt% reused	n/a
Ye et al., 2021 [77]	DGEBA-MHHPA CFRP	15 g	n/r	Benzyl alcohol/K_3_PO_4_ or NaOH	195 °C, 40 min; alkali:BnOH = 1:10 *w*/*w*	>90	Ester	OH, COOH	n/r	Clean surface; tensile strength ↓ 7.7% (K_3_PO_4_) and 4.4% (NaOH);
Si et al., 2022 [71]	DGEBA-MHHPA CFRP	2 g	n/r	Benzyl alcohol/NaOH	190 °C, 1 h	98.4	Ester	OH, COOH	n/r	Clean surface; tensile strength ↓ 5%;
Zhao et al., 2022 [85]	DGEBA-MTHPA neat resin	1.5 g	140.8	Ethanol/K_3_PO_4_ + 1 M HCl hydrolysis	120 °C, 3 h (alcoholysis) + 60 °C, 1 h (hydrolysis)	95.19 ± 3.68	Ester	OH, COOH	31.6 wt% reused; comparable strength	n/a
Min et al., 2025 [86]	DGEBA-MNA CFRP	1.55 g	n/r	β-phenethyl alcohol/TBD	190 °C, 2 h	100	Ester	OH, COOH	n/r	Graphitic structure preserved; tensile strength ↓ 7%;
Zhang et al., 2025 [87]	DGEBA-MTHPA powder	n/r	n/r	Ethanol/K_3_PO_4_	120 °C, 3 h; resin:ethanol:K_3_PO_4_ = 3:30:1 w/w/w	≈100	Ester	OH, COOH	22 wt% reused; tensile strength and Tg retained	n/a
Liu et al., 2025 [88]	DGEBA-MHHPA neat resin	n/r	164.7	BnOH/K_3_PO_4_	190 °C, 1 h	100	Ester	OH, COOH	Up to 30 wt% reused	n/a

Note: n/r = information not reported; n/a = not applicable; ↓ = decrease in the measured property, by the stated percentage.

Zhang et al. [78] developed a mild organocatalytic method for degrading DGEBA/MTHPA samples (40 × 10 × 4 mm^3^) using N-methyl-4-piperidinol (NHPP) in ethylene glycol (EG). Resin chips were mixed with EG and various organic bases under reflux conditions. Optimal parameters were identified as 180 °C for 1 h with an NHPP concentration of 0.2 mol/L, yielding 95.7% degradation. After reaction, the solid residue was filtered and washed, while EG and NHPP were recovered by vacuum distillation (120 °C, 1325 Pa) and successfully reused for at least three cycles, maintaining degradation yields usually above 90%. Characterization of the degradation products by GC, NMR, and ESI-MS confirmed the structural integrity of the recovered solvents and cleavage of ester crosslinks, leading to the formation of hydroxyl-rich compounds such as esterdiols and tetraalcohols. These intermediates have potential for reuse in new epoxy resins or in polyurethane foams. An example of the transesterification mechanism catalyzed by the organic base is shown in Figure 12.

The approach of Kuang et al. [79] is an atmospheric-pressure, mild-temperature method for decomposing resin and CFRP (DGEBA/HP) (Tg = 157 °C) using N-methyl-2-pyrrolidone (NMP) as solvent, ethylene glycol (EG) as co-reactant and the strong organic base TBD as a catalyst. The aprotic solvent NMP swells and plasticizes the cross-linked matrix, enhancing access to reactive sites, while TBD activates ester groups. EG then acts as the nucleophile that attacks and cleaves the activated ester linkages via transesterification. The synergistic interaction of these three components enables rapid and selective network degradation, achieving 95% degradation in 90 min at 170 °C. Comprehensive characterization using FTIR, NMR, SEM, and optical microscopy confirmed complete ester-bond cleavage and preservation of reclaimed fiber quality. The study also evaluated the potential reuse of DMPs as reactive components of a new epoxy formulation. Incorporating up to 20 wt% DMP allowed the resulting materials to retain tensile strength and modulus comparable to neat epoxy. However, DMA revealed decreasing Tg and rubbery modulus with higher DMP loadings, indicating plasticization effects [79].

The work of Shi et al. (2022) [93] also focuses on the degradation of neat epoxy resin (DGEBA/MHHPA) by a combination of EG/NMP solvent and TBD as catalyst (with the best results obtained for an EG/NMP mole ratio of 1:1.5 and TBD 0.3 mol/L at T = 160 °C). The study investigates the effect of different crosslinking densities (prepared by changing the molar ratio of DGEBA to MHHPA, resulting in Tg values of 46 °C to 90 °C) on degradation behavior. As expected, the crosslink density of the resin affects the degradation time, which increases from approximately 34 min for the lowest-crosslinked sample to approximately 253 min for the highest-crosslinked sample under the optimized EG/NMP ratio. The study primarily focused on the effects of crosslink density, solvent composition, and processing conditions on depolymerization kinetics [93].

In 2022, Mu et al. [69] developed a mild chemical recycling method for anhydride-cured epoxy resin formulated from DGEBA, glutaric anhydride (GA), and zinc acetylacetonate Zn(acac)_2_ (as an embedded transesterification catalyst). The process used mixed solvents consisting of 50% alcohol (EG or PG) and 50% aprotic solvent (DMF, NMP or DMSO). Degradation experiments containing 20 wt% cured epoxy resin (10 × 10 × 5 mm^3^) showed that the optimal solvent system was DMF/EG (50/50 wt%) at 150 °C for 180 min, even without a catalyst. Reduced-pressure distillation enabled recovery of solvents and yielded viscous depolymerized residues. The authors proposed a two-step mechanism: solvent diffusion first swells the epoxy network, enabling alcohol molecules to cleave ester bonds at elevated temperature. To accelerate the process, the catalysts TBD and Zn(acac)_2_ (0.3 mol/L) were introduced, reducing the required reaction time to 120 min with TBD, which is 60 min faster than uncatalyzed degradation [69].

The resulting DMPs were blended into fresh resin at 5 wt% and 20 wt%. Tensile testing showed that increasing DMP content reduced modulus and ultimate tensile stress by 6% and 26%, respectively, while raising ultimate elongation by 41% at 20 wt%, indicating a plasticizing effect. The study also evaluated a CFRP laminate (18 × 18 × 2 mm^3^) under the optimized catalyst-free conditions (DMF/EG 50/50, 150 °C). Clean carbon fibers were fully recovered after 160 min, and tensile testing revealed negligible differences between virgin and reclaimed fibers, confirming that the method preserves fiber quality [69].

Xu Zhao et al. [89] proposed a two-step process to digest MTHPA-cured bisphenol-A epoxy resin powder (ground to pass a 60-mesh sieve, i.e., particle sizes below approximately 250 μm; Tg = 112 °C): a first swelling step in dichloromethane, then a reaction with NaOH in ethylene glycol at 150 °C for 1 h, resulting in 100% depolymerization. The degradation products were separated into a solid carboxylate fraction (DEP-COO^−^) and a liquid hydroxyl-rich fraction (DEP-OH) by ethanol-induced precipitation followed by filtration of solid particles (solid DEP-COO^−^), while the filtrate was concentrated and poured into water to induce phase separation, yielding an oil-phase DEP-OH. These intermediates were subsequently converted into functional epoxy building blocks: DEP-OH was transformed into a recycled epoxy monomer (EDEP) via reaction with epichlorohydrin, and DEP-COO^−^ was converted into an anhydride curing agent (ADEP) by acidification with 18 wt% HCl under reflux at 80 °C for 2 h to obtain DEP-COOH, followed by addition of acetic anhydride under a nitrogen atmosphere and subsequent removal of acetic anhydride to obtain ADEP. The yields of both the epoxy monomer and curing agent exceeded 89% and 90%, respectively. New epoxy thermosets were synthesized exclusively from the recycled epoxy monomer and curing agent, without the addition of virgin components. Among the tested formulations, the system with an EDEP:ADEP ratio of 10:9 showed the best overall performance, with a tensile strength of 83 MPa, a glass-transition temperature of 106 °C, and an initial thermal decomposition temperature of 299.3 °C. Life-cycle and economic analyses highlighted the sustainability of the process, estimating a reduction of 963.9 kg CO_2_ per ton of recycled epoxy thermoset and a net profit of approximately $111.9 per ton, excluding additional savings from avoided waste disposal [89].

In a subsequent study, Xu Zhao et al. [94] developed an in situ degradation-foaming process to directly convert epoxy thermosets into polyurethane foam. A self-separating catalytic degradation system (K_3_PO_4_/PEG) was employed to degrade MTHPA-cured DGEBA epoxy thermosets. In this process, PEG acts both as a degradation solvent and as the main component of PU foam. After degradation, K_3_PO_4_ was removed by simple filtration, and the degradation solution (DS) was directly used to produce PU foam without additional separation. With the rigid epoxy skeleton retained in the degradation products, the resulting polyurethane foam exhibited good mechanical properties, good thermal insulation performance, low water absorption, and good thermal stability.

Tian et al. [90] reported a non-toxic glycolysis method for degrading anhydride-cured epoxy thermosets (DGEBA cured with MTHPA) in which a deep eutectic solvent (DES) prepared from potassium carbonate (K_2_CO_3_) and ethylene glycol (EG) was used. The DES (molar ratio K_2_CO_3_:EG = 1:20, 0.12 g mL^−1^) was capable of nearly complete degradation within 3 h at 180 °C under ambient pressure. FTIR and ^1^H NMR indicated selective ester bond cleavage via transesterification, as confirmed by the disappearance of anhydride-derived ester linkages and the formation of hydroxyl-terminated DGEBA tetra-alcohol derivatives, while the aromatic backbone remained intact. The system also reclaimed 100% of carbon fibers from epoxy composites, and partial recovery of K_2_CO_3_ was achieved through ethanol-assisted precipitation.

#### 2.3.4. Aminolysis

In aminolysis, primary or secondary amines act as nucleophiles toward ester bonds, leading to the formation of amide groups and hydroxyl-terminated epoxy fragments. Compared with hydrolysis and alcoholysis, aminolysis is typically faster and thermodynamically favored due to the high stability of the amide linkage, rendering the process largely irreversible [66,67,70,95].

Aminolysis of esters is a reaction in which an ester reacts with ammonia or an amine to form an amide and an alcohol. The already reported study of K. El Gersifi, G. Durand, G. Tersac [92] reports that monoethanolamine exhibits markedly higher reactivity than diethylene glycol under the investigated conditions, enabling efficient solvolysis even in the absence of added catalysts and at lower temperatures. Ethanolamines have previously been employed for the solvolysis of various polymers, including PET, polyurethanes, and unsaturated polyesters, where the amine functionality can act as an intrinsic transesterification catalyst.

Zhao et al. [66] investigated the digestion of an MTHPA-cured epoxy resin with a glass transition temperature (Tg) of 126.3 °C, first pretreated in dichloromethane (DCM) at a 2:5 (*w*/*w*) ratio for 48 h to swell the material prior to degradation. The pretreated resin was then placed in a microwave reactor and mixed with diethylenetriamine (DETA) at a 5:2 (*w*/*w*) DETA-to-sample ratio. The authors reported that the degradation degree increased with temperature (110 → 130 °C) and reaction time (20 → 50 min), while larger resin loadings reduced the degradation efficiency. Under optimal conditions—130 °C for 50 min with a MER/DETA ratio of 2:5 (*w*/*w*)—a degradation degree of 99% was achieved.

The decomposed resin was characterized by FTIR, NMR, GPC, and elemental analysis. FTIR confirmed that the polymer backbone remained largely intact, while the intensity of ester-group signals sharply decreased. Concurrently, the emergence and growth of amide and hydroxyl peaks indicated aminolysis of ester bonds. NMR corroborated these findings, revealing a marked reduction in ester peaks and the appearance of a small amide-group signal. Elemental analysis further confirmed an increase in nitrogen content, consistent with amide formation during degradation. The authors also blended the degradation products directly with the uncured epoxy monomer. The degradation liquid, containing oligomers and excess DETA, served as a curing agent without prior separation, representing a closed-loop reuse strategy. Mixtures containing 9–40 wt% degradation solution were evaluated, and the formulation with 30 wt% yielded the highest Tg (146.4 °C), surpassing that of the neat resin (134.7 °C) [66].

In a follow up study [70], the recovered carbon fibers were characterized by SEM, XPS, Raman spectroscopy, and tensile testing. The fibers exhibited clean surfaces with no observable defects and retained nearly their original structure and chemistry, showing only minor reductions in tensile strength and modulus (4% and 5%, respectively). The authors also evaluated the reuse of both recovered resin fragments and recycled solvent. Incorporating DMP into fresh epoxy resin resulted in substantial decreases in flexural strength, falling to 50% with only 10% DMP content. Recovered MEA contained 85–90 vol% of MEA and maintained 98% of the degradation efficiency in a second reaction cycle, though efficiency declined to 80% upon a third use [70].

Shao et al. [67] developed a catalyst-free aminolysis method for recycling anhydride-cured epoxy resins (DGEBA cured with HHPA, Tg =148 °C) using aminoethanol (EA) under atmospheric pressure. Cured resin samples were immersed in EA at a 1:4 weight ratio and heated at 120–160 °C, with complete degradation achieved after 4 h at 160 °C. FTIR analysis confirmed the disappearance of ester bonds and the formation of amide bonds, demonstrating full cleavage of the network. NMR further verified the presence of expected degradation products such as BPA-OH (a polyhydric compound derived from the epoxy residue, whose structure is mainly based on the bisphenol-A (BPA) segment of the epoxy resin) and HHPA-OH (a white crystalline amide diol compound derived from the curing agent residue). Therefore, BPA-OH contains multiple hydroxyl (-OH) groups, whereas HHPA-OH contains both amide (-CONH-) and hydroxyl (-OH) groups. The results indicated that the epoxy network was successfully digested into high-purity monomers without requiring catalysts or high-pressure conditions. Regarding separation, the process did not isolate “alcohols” from “amides” as functional groups. Instead, after degradation, excess EA was removed by reduced-pressure distillation at 80–110 °C. The recovered monomers were then repurposed into new materials with strong performance, showcasing the method’s upcycling potential. BPA-OH was used to synthesize a polyurethane coating that exhibited a Tg of 88.9 °C, tensile strength of 78.99 MPa, excellent adhesion (5B), high hardness (up to 8H), and strong solvent resistance. HHPA-OH was converted into a dimethacrylate ester (DME-HHPA) and used as a crosslinker for 3D printing resins.

**Table 3:** Glycolysis routes for epoxy thermoset solvolysis.

Reference	Digested System	Size/ Weight	Tg (°C)	Solvent /Catalyst	Optimal Conditions	Degrad. (%)	Cleaved Bonds	Restored Functionality	Recyclable DMP	Recovered CF Quality
El Gersifi et al., 2006 [92]	DGEBA-HP neat resin	Granular	132	DEG/TBT	245 °C, 1.5–3 h	100	Ester	OH (polyol)	In PU foams	n/a
Yang et al., 2014 [91]	DGEBA-MTHPA CFRP	1.92 cm^3^	n/r	PEG200/NaOH	200 °C, 4 h	84–93	Ester	n/r	n/r	Tensile strength ↓ 4–6%; slight oxidation
Zhang et al., 2018 [78]	DGEBA-MTHPA neat resin	1.60 cm^3^	n/r	EG/N-methyl-4-piperidinol	180 °C, 3 h	99.7	Ester	OH	n/r	n/a
Kuang et al., 2018 [79]	DGEBA-HP neat resin & CFRP	125 mm^3^ 13.9 g	157	EG/NMP/TBD	170 °C, 90 min	95	Ester	OH	≤20 wt% reused in new epoxy	Unchanged properties
Shi et al., 2022 [93]	DGEBA-MHHPA neat resin	0.50 cm^3^	46	EG/NMP/TBD	160 °C, 34 min	100	Ester	n/r	n/r	n/a
Mu et al., 2022 [69]	DGEBA-GA neat resin & CFRP	10 × 10 × 5 mm^3^ (neat resin); 18 × 18 × 2 mm^3^ (CFRP)	n/r	DMF/EG	150 °C, 160 min	≈100	Ester	OH	≤20 wt% reused in new epoxy	Unchanged properties
Zhao et al., 2023 [89]	DGEBA-MTHPA neat resin	3 g powder	112	DCM swelling, EG/NaOH	150 °C, 1 h	100	Ester	OH, COO^−^	>80% of new epoxy thermoset derived from degradation products	n/a
Zhao et al., 2023 [94]	DGEBA-MTHPA neat resin	2 g powder	n/r	PEG/K3PO4	130 °C, 1 h	95	Ester	OH, COO^−^	Degradation solution directly used in PU foam	n/a
Tian et al., 2025 [90]	DGEBA-MeTHPA neat resin & CFRP	≈2.7 g neat resin, CFRP n/r	n/r	Deep eutectic solvent	180 °C, 3 h	≈100	Ester	OH, COO^−^	n/r	No major surface defects; tensile strength ↓ 36.6%

Note: n/r = information not reported; n/a = not applicable; ↓ = decrease in the measured property, by the stated percentage.

In a recent study, Yang et al. [95] developed an anhydride-cured epoxy system (DxBVy/NMA) incorporating dual dynamic covalent bonds (ester and imine) by combining a bio-based Schiff base epoxy monomer (BV-EP) with DGEBA and curing with nadic methyl anhydride (NMA). The incorporation of imine bonds was designed to introduce additional cleavable sites within the crosslinked network while maintaining satisfactory thermomechanical properties (Tg = 150 °C for the optimized D5BV5/NMA formulation). The optimized formulation of the epoxy resin system achieved complete degradation in aminoethanol (EA) at 160 °C within 25 min, which represents high efficiency under mild conditions. FTIR and NMR analyses confirmed cleavage of both ester and imine bonds and generation of amide and hydroxyl functionalities. In addition, the digestion process showed the reclamation of CFs from CFRP by fully degradation of the epoxy resin matrix. SEM, XRD, and Raman analyses verified that rCFs did not show any structural damage. The key novelty of this work relies on the construction of a dual dynamic network to enhance degradability of the matrix while preserving mechanical properties, allowing rapid, non-destructive composite recycling under relatively mild conditions.

#### 2.3.5. Acetolysis

Acetolysis approaches rely on strongly acidic, acylating media and are characterized by low selectivity, resulting in the simultaneous cleavage of ester, ether, and even aryl–carbon bonds. These harsh conditions and poor selectivity have confined such methods largely to proof-of-concept degradation studies, preventing their development into scalable or value-preserving recycling strategies [68].

Lei et al. [68] demonstrated that an anhydride-cured epoxy resin can be efficiently degraded using a mixture of p-toluenesulfonic acid and acetic anhydride (in a 3:2 weight ratio). Optimal conditions were identified as 2 h of reaction at 130 °C with a cleavage-agent-to-resin ratio of 20:1. Characterization by IR, ^1^H NMR, GC–MS and GC revealed that degradation proceeds through multiple bond-cleavage steps, including the breaking of ester linkages, benzene–carbon bonds and carbon–oxygen ether bonds. p-Toluenesulfonic acid (p-TSA) protonates ester carbonyls formed in the epoxy–anhydride network. Acetic anhydride acts as a reactive acylating medium, enabling ester scission via acetolysis. The reported cleavage of benzene–carbon bonds indicates carbocation-type pathways (Friedel–Crafts-like rearrangements or fragmentation), characteristic of superacid-strength media. Multiple bond types (ester, ether, aryl–C) are attacked, indicating low selectivity but high aggressiveness.

Table 5 summarizes the principal mild solvolysis routes for epoxy thermoset depolymerization, highlighting their operating conditions, degradation performance, and circularity potential. Overall, alcoholysis, glycolysis and aminolysis generally enable efficient ester bond cleavage under milder reaction conditions than hydrolysis, whereas each route presents distinct advantages and limitations regarding reaction severity, product recovery and process sustainability.

## 3. Safety, Health, and Environmental Considerations in Chemical Recycling Processes

Any newly developed solvolysis route that shows potential for industrial application should be assessed from a life cycle perspective, so that no environmental hotspots or bottlenecks are overlooked. The assessment should cover the whole chain, from the waste material fed into the process to the recovered products (fibers and resin). It should also consider whether solvents, co-reactants and catalysts come from renewable sources, the impacts of their manufacturing, recycling and/or disposal processes, the costs and security of their supply, and many other aspects that are normally included in a life cycle assessment (LCA). On the other hand, the depth of this analysis, and therefore its complexity, should match the stage of research and development. It can range from a broad initial screening of reactions run on a milligram scale to detailed analyses that include the aforementioned life cycle considerations, carried out at pilot scale (kilogram quantities). Depending on the key parameters identified, qualitative and quantitative metrics can be applied at each level of technological development. This tiered approach was proposed by McElroy et al. [96] within the CHEM21 project (Chemical Manufacturing Methods for the 21st Century Pharmaceutical Industries), which aimed to make the pharmaceutical industry more sustainable. According to McElroy et al. [96], the Zero Pass assessment covers yield, conversion and selectivity, atom economy (AE) and/or reaction mass efficiency (RME), the choice of solvents, and the health and safety hazards of all reactants. It is intended for the discovery stage, where large numbers of screening reactions are carried out on a small scale. Its purpose is to flag any use of highly hazardous substances and to give a first estimate of how efficient the route is. As such, it shows only that a reaction has no serious issues and not that it is green. Promising reactions then move on to the First, Second and Third Passes.

The Second and Third Passes are for reactions and pathways that have reached pilot scale or beyond. The Second Pass uses data from 2–10 L batch reactions, or from continuous runs that produce more than 1 kg of product in less time than the bench-scale reaction. It revisits MI/PMI, now including solvent recovery and reuse. It also adds metrics that would take too much time, or make little sense, at bench scale: catalyst turnover number and recoverability, space–time yield, renewables intensity, the upstream impact of preparing the reagents and catalysts, a process safety assessment, a streamlined life cycle assessment (LCA) and an estimate of raw-material costs. The Third Pass is a desk exercise, carried out if the Second Pass results are promising, to assess whether the process is feasible at industrial scale. It covers a full energy analysis, including upstream and downstream contributions such as catalyst preparation, work-up, solvent recovery and waste treatment. It also covers the recovery and recycling of solvents, reagents and by-products, including energy recovery, and the nature and fate of the waste, expressed as waste intensity and waste percentage.

Given the scope of this review, the types of process and product involved, and above all the limits of the available data, which come entirely from gram-scale experiments, the assessment was carried out essentially at the Zero/First Pass level.

**Table 4:** Aminolysis and acetolysis routes for epoxy thermoset solvolysis.

Reference	Digested System	Size/ Weight	Tg (°C)	Solvent/ Catalyst	Optimal Conditions	Degrad. (%)	Cleaved Bonds	Restored Functionality	Recyclable DMP	Recovered CF Quality
Zhao et al., 2019 [66]	DGEBA-MTHPA neat resin	2 g	126.3	DCM pretreatment/DETA	130 °C, 50 min	99	Ester	CONH, OH	9–40 wt% reused	n/a
Zhao et al., 2022 [70]	DGEBA-MTHPA CFRP	1 g	127	MEA/KOH	160–163 °C, 60 min	99	Ester	CONH, OH, COO^−^	Up to 10 wt% reused	Clean surface; tensile strength ↓ 4%; Young’s modulus ↓ 5%;
Shao et al., 2023 [67]	DGEBA-HHPA neat resin	0.18 g	148	Aminoethanol	160 °C, 4 h	100	Ester	OH, CONH	BPA-OH → PU coating; HHPA-OH → 3D-print crosslinker	n/a
Yang et al., 2025 [95]	D5BV5/NMA neat resin & CF-D5BV5/NMA CFRP	0.25 g neat resin, CFRP n/r	151	Aminoethanol	160 °C, 25 min, resin:EA = 1:5	100	Ester; imine	OH, CONH, NH_2_	High-purity BAPF recovered	No damage
Lei et al., 2016 [68]	DGEBA-MTHPA neat resin	0.2 g powder	n/r	p-TSA/acetic anhydride	130 °C, 2 h	n/r	Ester, ether, aromatic C–C	n/r	n/r	n/a

Note: n/r = information not reported; n/a = not applicable; ↓ = decrease in the measured property, by the stated percentage.

**Table 5:** Comparative assessment of mild epoxy thermoset depolymerization strategies.

Solvolysis Route	Typical Temperature (°C)	Pressure Requirement	Main Bonds Cleaved	Degrad. (%)	Restored Functionality	Circularity Potential
Hydrolysis	190–405	Medium–High	Ester, ether	97.7–100	OH, COOH	Moderate
Alcoholysis	120–285	Low–Medium	Ester	>90–100	OH, COOH, OCH_3_	High
Glycolysis	130–245	Atmospheric	Ester	84–100	OH	High
Aminolysis	160–163	Atmospheric	Ester	99–100	OH, CONH_2_	Very high
Acetolysis	130	Atmospheric	Ester, ether, aromatic C–C	n/r	Various aromatics	Emerging

Adapting this approach to such a different type of process requires careful consideration. As discussed above, conversion of the cross-linked resin into degradation products is almost always complete. Without additional co-reactants, as in hydrolysis under near-critical or supercritical conditions, this is achieved only under harsher conditions of temperature and pressure. Otherwise, it requires co-reactants that are consumed during the reaction, specific catalysts, organic solvents, or a combination of these (e.g., DETA or monoethanolamine/aminoethanol in aminolysis [66,67,70,95]). In all cases, solvolysis does not lead to a single target product. The network can be broken down into molecules with a molecular weight comparable to that of the monomers, into larger oligomers, or into fragments of varying size and cross-link density (e.g., BPA-OH and HHPA-OH obtained by aminolysis in Shao et al. [67], and soluble depolymerized epoxy oligomers obtained in Mu et al. [69]). Without a single target product, the classic metrics of yield and selectivity cannot be applied. For the same reason, atom economy (AE), which measures reaction efficiency as the proportion of reactant atoms that end up in the final product, is of no practical use. Reaction mass efficiency (RME), defined as the mass of isolated product(s) divided by the total mass of reactants, can only be calculated once the isolated product has been defined. In some studies, this is the recovered fiber alone; in others, it is both the fibers and the degraded matrix. This greatly reduces the number of studies that can be compared. In addition, small-scale experiments are often run in a large excess of solvent for ease of handling, so mass-based metrics that include the solvent, such as MI/PMI, are dominated by its contribution, which masks differences in the underlying chemistry.

On the other hand, the health and safety hazards of the solvents and reactants can already be assessed at the preliminary screening stage, using their hazard statements (H-statements) under the Globally Harmonized System of Classification and Labelling of Chemicals (GHS) [96].

In the CHEM21 Zero Pass, any substance carrying one of the most severe H-statements receives a red flag. These are: explosive (H200–H203), risk of explosive thermal runaway (H240), fatal if swallowed, in contact with skin or if inhaled (H300, H310, H330), may cause cancer (H350), may damage fertility or the unborn child (H360), and harmful to the ozone layer (H420). The First Pass extends this to a wider range of H-statements, which generate red, yellow or amber flags [96].

For solvents, the CHEM21 solvent selection guide combines H-statements with physical properties such as flash point, auto-ignition temperature and boiling point. It assigns separate safety, health, and environment scores and an overall ranking (recommended, problematic, hazardous) [97] (Figure 13). These scores describe the intrinsic hazards of each substance, independently of the conditions under which it is used. The analysis presented here should therefore be regarded as a comparative hazard screening rather than a risk assessment. Hazards arising from the process itself, such as high temperature and pressure, have been assessed separately. Finally, the absence of an H-statement does not necessarily mean the absence of a hazard. Any substance without adequate safety data should be treated as potentially harmful.

### 3.1. Health and Safety Hazards of the Solvents and Reactants

The safety assessment of chemicals is based on their flammability, which is measured by the substance’s flash point (FP), combined with the boiling point (BP) when FP < 24 °C. The lowest score, 1, belongs to solvents with FP above 60 °C, which is associated with a minimum flammability risk, while solvents with lower flash points obtain higher scores, up to 7. One extra safety score (+1) is assigned to chemicals that show each of the following characteristics: chemicals with auto-ignition temperature (AIT) lower than 200 °C; chemicals that accumulate electrostatic charge, if resistivity is higher than 10^8^ Ω m; and chemicals that can form explosive peroxides, as indicated by the hazard statement EUH019. Moreover, solvents with a high energy of decomposition (>500 J g^−1^) are scored 10.

When more than one H-phrase is associated with the chemical, the score corresponding to the highest hazard is assigned. In addition, solvents with boiling points below 85 °C are assigned an extra penalty to reflect their increased volatility that underlies higher inhalation risks. Score 1 is attributed to the chemicals that do not have H-phrases associated with health risks and score 5 to solvents with incomplete toxicological data.

Environmental scores were assigned on three levels (3, 5 and 7), considering both ozone depletion potential and solvent boiling point. The highest score (7) was attributed to solvents with boiling points above 200 °C or below 50 °C, as low-boiling solvents tend to generate volatile organic compounds (VOCs), while high-boiling solvents are difficult to recover and recycle. Solvents that contribute to ozone depletion were automatically assigned a score of 10, identifying them as the most environmentally hazardous compounds. As for health risks, solvents that are not of environmental concern are scored 1 and solvents without full environmental toxicity data are given a score of 5 by default. Based on the described scoring methodology and the information provided in the safety data sheets of the solvents used in studies on the recycling of epoxy resin systems that were reviewed, Table 6 summarizes the scores assigned to each solvent. These scores describe the intrinsic hazards of each substance and are therefore independent of the conditions under which it is used: water and methanol receive the same scores whether they are used at atmospheric pressure or under near-critical or supercritical conditions. High temperature and pressure are process hazards and are considered separately in Table 8 and Figure 14. Since exposure, quantities, engineering controls and accident probability are not considered as the data derived from gram-scale experiments, the analysis presented here is a comparative hazard screening rather than a risk assessment. A color code was associated with the scoring: green for 1–3, yellow for 4–6, and red for 7–10.

The final ranking is not made on the basis of the sum of the SHE scores or any average, because these approaches can underestimate a major issue in one of the categories, but is based on the most stringent combination: “hazardous” when one score is equal to or higher than 8, or the solvent has two “red” scores; “problematic” when one score is equal to 7 or there are two “yellow” scores; “recommended” in any other case. According to the scores and ranking approach, only three solvents are classified as recommended: water, ethanol and ethylene glycol. As described by Prat et al. in their paper [97], the ranking can be revised after expert discussion. For example, methanol was reclassified as Recommended in the CHEM21 reference after expert discussion, acknowledging its bio-sourced availability and relatively manageable toxicity profile. While we recognize that such re-evaluations are meaningful, they are best addressed through an LCA approach, which requires regional and local data. We therefore refrain from incorporating these expert adjustments into our scoring system.

To obtain a more comprehensive evaluation of the overall SHE performance of the chemical recycling systems, we applied the same methodology to co-reactants and catalysts, and the results are reported in Table 7. As expected, since almost all are non-flammable solids, the safety score is not a differentiating factor. The decisive variables are corrosivity (H314, driving H = 7) and aquatic toxicity (H410/H411/H412, driving E = 5–7). The inorganic carbonate and phosphate bases (K_2_CO_3_, K_3_PO_4_) perform best precisely because they avoid both. N-methyl-4-piperidinol has a slight environmental penalty (E = 5) due to its boiling point and is classified as problematic. The inorganic bases, KOH and NaOH, are classified as problematic primarily because of their H314 classification (severe skin burns and eye damage), which yields a health score of 7, while their safety and environmental scores remain relatively low (S = 1, E = 3). These health hazards are well known and can be managed in industrial settings with appropriate safety controls.

Table 8 and Figure 14 provide a comparative overview of the SHE and operational characteristics of hydrolysis, alcoholysis, glycolysis, aminolysis, and acetolysis approaches used for the degradation of anhydride-cured epoxy resins. Almost all the reported approaches achieve high degradation efficiencies, typically above 90% (84–100%, Table 8). Solvolysis is effective in degrading the epoxy matrix, regardless of the specific route selected and provided that the reaction conditions are optimized. This factor cannot therefore be considered a discriminating criterion. In Table 8, degradation (%) is given as reported in each study. In most studies on CFRP, it is calculated from the resin content of the composite and the weight loss measured at the end of the process, on the assumption that all the weight loss is due to resin removal. The value should therefore be regarded as an upper estimate, since any loss of fiber mass, such as removal of the sizing, fiber oxidation or loss of fiber fragments, is also counted as degradation.

Although the degraded resin fraction and the quality of the recovered fibers are described in different ways and in varying depth across the studies, some observations can still be drawn by considering the reaction temperature, the reaction time, and the solvent and co-reactant mass per unit mass of resin, together with the intrinsic hazards of the chemicals involved (Table 6 and Table 7). As stated previously, typical industrial and economic considerations are not taken into consideration, given their regional dependency and the fact that most of the reported data relate to laboratory-scale experiments.

For the representation of the overall SHE positioning, a score of 7 was assigned to the *Hazardous* category (red), a score of 5 was assigned to the *Problematic* category (yellow) and a score of 3 to the *Recommended* category (green); a score of 3 was also assigned to entries where no co-reactant or catalyst was used. When solvent mixtures were used, the associated color was that of the most hazardous component. To assess the impact of materials in the solvolysis process, only the solvent mass per unit mass of resin or CFRP was considered, as catalysts and co-reactants are typically used in much smaller quantities (about 1–10% of the solvent mass). These ratios are reported in Table 8; depending on the study, they refer either to the mass of resin or to the mass of CFRP, so they are indicative and not strictly comparable. Although small relative to the solvent, co-reactant loadings can still be significant relative to the resin processed, and they are therefore assessed separately in Section 3.2.

Figure 14 shows reaction temperature on the x-axis and reaction time as bubble size. As a simple indication of energy demand, we refer to the temperature bands of the CHEM21 First Pass (McElroy et al. [96]): reactions between 0 and 70 °C receive a green flag, those outside this range but within −20 to 140 °C a yellow flag, and those outside this wider range a red flag. Only processes operating at atmospheric pressure are compared. Processes operating under pressure are listed in Table 8 but are not placed on the same axis, because pressure introduces hazards and energy demand that temperature alone does not capture. Figure 14 shows that almost all the atmospheric-pressure approaches operate above 140 °C and would therefore receive a red energy flag in the CHEM21 First Pass; only alcoholysis in ethanol/K_3_PO_4_ at 120 °C, glycolysis in PEG 400 (130 °C), aminolysis in DETA/DCM and acetolysis (both at 130 °C) fall below this threshold. No single approach combines a low combined SHE score with a low reaction temperature and a short reaction time. Glycolysis in ethylene glycol alone comes closest to this target; however, this study was conducted on neat resin rather than on CFRP [78]. These observations indicate that SHE performance and process mildness have not yet been jointly optimized in the solvolysis of carbon fiber-reinforced composites.

The pressurized routes listed in Table 8 (marked †) include hydrolysis in near-critical or supercritical water that uses no organic solvent and, in the studies listed, no co-reactant, so it scores best on intrinsic chemical hazard (Table 6 and Table 8). This advantage comes at the cost of temperatures of 280–405 °C and pressures ranging from several MPa to above the critical pressure of water (22.1 MPa). Subcritical alcoholysis also operates under pressure; for example, methanol at 210 °C and 4.5 MPa [76]. These conditions require pressure-rated reactors, increase capital costs and energy demand, and introduce process hazards such as stored pressure energy and high-temperature fluids, which the intrinsic SHE scores do not capture. Whether the absence of organic solvents outweighs these drawbacks can only be judged with process-level data on energy demand and heat recovery, which are not available for the studies reviewed.

**Table 6:** SHE hazard screening (intrinsic properties) of the solvents used for the recycling of anhydride-cured epoxy resin systems.

Solvent	Scoring Basis	Safety	Health	Environ.	Ranking
Water [72,73,74,81,82,84]	S: non-flammable; H: no H-statements; E: water baseline	1	1	1	Recommended
DEG [92]	S: FP 138 °C; H: H302, H373; E: BP 245 °C	1	2	7	Problematic
Methanol [75,76]	S: H225, FP 11 °C; H: H370; E: BP 65 °C	4	7	5	Problematic
Acetic anhydride [68]	S: H226, FP ~49 °C; H: H314; E: BP ~139 °C	3	7	3	Problematic
Ethanol [85,87]	S: H225, FP 13 °C; H: H319, BP < 85 °C (+1); E: BP 78 °C	4	3	3	Recommended
EG [69,78,79,89,90,93]	S: FP 111 °C; H: H302, H373; E: BP 197 °C	1	2	5	Recommended
NMP [79,93]	S: FP 91 °C; H: H315, H319, H360D, H373; E: BP 202 °C	1	9	7	Hazardous
β-Phenethyl alcohol [86]	S: FP 96 °C; H: H302, H312, H332; E: BP 219 °C	1	2	7	Problematic
DCM [66,89,94]	S: non-flammable; H: H315, H319, H336, H351, H373; E: BP 40 °C	1	7	7	Hazardous
DETA [66]	S: FP 94 °C; H: H302, H311, H314, H317, H330, H335; E: BP 207 °C	1	9	7	Hazardous
Benzyl alcohol [71,77,88]	S: FP 93 °C; H: H302, H317, H319, H332; E: BP 205 °C	1	2	7	Problematic
EA/MEA [67,70,92,95]	S: FP 85 °C; H: H290, H302, H311, H314, H331; E: BP 171 °C, H412	1	7	5	Problematic
PEG 400 [94]	S: FP 238 °C; H: no GHS classification; E: BP/decomp. > 250 °C	1	5	7	Problematic
PEG 200 [91]	S: FP > 60 °C; H: no GHS classification; E: BP/decomp. > 200 °C	1	5	7	Problematic
DMF [69]	S: FP 58 °C; H: H226, H312, H319, H331, H360D, H373; E: BP 153 °C	3	9	5	Hazardous

Scores and ranking follow the CHEM21 solvent selection guide [97]; they describe intrinsic hazards and do not depend on operating conditions. Color code: green, 1–3; yellow, 4–6; red, 7–10. S = safety; H = health; E = environment; FP = flash point; BP = boiling point. DEG = diethylene glycol; EG = ethylene glycol; NMP = N-methyl-2-pyrrolidone; DCM = dichloromethane; DETA = diethylenetriamine; EA/MEA = ethanolamine (monoethanolamine); PEG = poly(ethylene glycol); DMF = N,N-dimethylformamide.

**Table 7:** SHE scores of the co-reactants and catalysts used in the reviewed studies.

Co-Reactant	Scoring Basis	Safety	Health	Environ.	Ranking
K_2_CO_3_ [90]	S: non-flammable solid; H: H315, H319, H335; E: no H4xx, non-volatile solid	1	3	3	Recommended
KOH [70,73,76]	S: non-flammable solid, H290; H: H314; E: no H4xx, non-volatile solid	1	7	3	Problematic
NaOH [71,77,89,91]	S: non-flammable solid, H290; H: H314; E: no H4xx, non-volatile solid	1	7	3	Problematic
K_3_PO_4_ [77,85,87,88,94]	S: non-flammable solid; H: H318, H335; E: no H4xx, non-volatile solid	1	4	3	Recommended
HPW [72]	S: non-flammable solid; H: H302, H314; E: H411	1	7	7	Hazardous
p-TSA [68]	S: no controlling flammability trigger; H: H314, H335; E: H412	1	7	5	Problematic
TBD [69,79,86,93]	S: no controlling flammability trigger; H: H314; E: no H4xx, insufficient data (default 5)	1	7	5	Problematic
N-Methyl-4-piperidinol [78]	S: FP 112 °C; H: H315, H318, H335; E: BP 200 °C	1	4	5	Problematic
Zn(acac)_2_ [69]	S: non-flammable solid; H: H317, H319; E: H410	1	3	7	Problematic
TBT [92]	S: H226, FP 42 °C; H: H318; E: BP > 200 °C	3	4	7	Problematic
H_2_SO_4_ [73]	S: non-flammable liquid, H290; H: H314; E: BP ~335 °C	1	7	7	Hazardous

Scores and ranking follow the methodology of the CHEM21 solvent selection guide [97], applied to intrinsic hazards. Color code: green, 1–3; yellow, 4–6; red, 7–10. S = safety; H = health; E = environment; FP = flash point; BP = boiling point. HPW = phosphotungstic acid; p-TSA = p-toluenesulfonic acid; TBD = 1,5,7-triazabicyclo [4.4.0]dec-5-ene; Zn(acac)_2_ = zinc acetylacetonate; TBT = titanium(IV) butoxide.

### 3.2. Assessment Against Additional Criteria

To extend the comparison beyond the intrinsic hazards of the chemicals, the studies listed in Table 8 were also assessed against the additional criteria proposed by the CHEM21 First Pass, as far as the reported data allow (Table 9) [96]. Each criterion receives a green, amber, yellow, or red flag.

The use of catalysts/co-reactants was flagged green when the loading was at most 0.1 g per g of resin or CFRP, and amber above this value. This threshold is our own assumption, as the reported data do not allow a comparison on a molar basis (e.g., whether the use is catalytic or stoichiometric). It is therefore used only to indicate whether a process contributes significantly to the consumption of additional chemicals.

Critical elements were flagged amber when the co-reactant contains an element included in the 2023 EU list of critical raw materials [98], which in this dataset applies only to phosphorus (K_3_PO_4_); the years-to-depletion criterion of the original toolkit was not applied.

Reflux was flagged red when the reaction temperature is within 5 °C of, or above, the atmospheric boiling point of the solvent, and green when it is at least 5 °C below; this criterion does not apply to processes run under pressure in closed reactors. All the reported processes are carried out in batch and therefore receive an amber flag.

Solvents listed as Substances of Very High Concern and restricted under REACH (NMP and DMF) were flagged red, and DCM, whose use is restricted in specific applications, amber. All the chemicals involved are commercially available and would receive a green flag for availability, so this criterion is not shown. Work-up was not assessed, because most studies do not describe the separation, washing and recovery steps in sufficient detail.

Table 9 shows that none of the reported routes performs well against all the First Pass criteria. All the processes are run in batch, and all except five, which operate between 120 and 130 °C, receive a red energy flag. In most studies, the co-reactant is used at 0.15–1.9 g per g of resin or composite processed, which increases the amount of chemicals to be recovered or disposed of. All these issues are expected for processes at laboratory bench scale. The use of NMP and DMF adds a regulatory concern to glycolysis routes that already score poorly in Table 6. Glycolysis in ethylene glycol alone again stands out, with no critical elements, no chemicals of concern and no reflux, but its reaction temperature (180 °C) still results in a red energy flag. Routes combining a low SHE score, a catalytic co-reactant loading and a moderate temperature are therefore still lacking and should be the target of future development before assessment at higher Pass levels becomes possible. In this discussion, credits for recovered materials were not applied when evaluating the environmental performance of the solvolysis processes. Although most studies report that the reclaimed fibers are clean and retain their mechanical performance (usually tensile strength and modulus), this does not automatically qualify them for credits for avoided virgin-material production, since other key properties, such as fiber length or surface condition, may change significantly. Similarly, credits for recovered resin-derived products or other co-products, as well as for avoided waste treatment, should be granted only where reuse has been demonstrated, which was not always investigated.

### 3.3. Scale-Up Considerations

Scaling up these processes involves technical and environmental challenges, including reactor design, solvent recovery rates, and heat and mass transfer, as detailed in Table 10. A direct comparison of LCA data across the chemical recycling processes investigated in this work is difficult, because the available data differ considerably in process maturity and scale [99,100,101,102,103]. Some routes, such as acetolysis and certain alcoholysis processes, are more advanced and supported by explicit process models and techno-economic assessments [102,104,105]. Most other routes have been studied only at laboratory scale. The future performance of the different solvolysis processes therefore cannot be projected from laboratory data alone [100]. On energy and reactor design, low-temperature processes have a smaller environmental footprint than high-temperature processes [103,104]. Even so, using renewable energy sources is essential for the environmental performance of any solvolysis process [103]. Another key step in scaling up is moving from batch to continuous operation, which improves energy efficiency and throughput for all process routes [106,107]. On-site solvolysis is effectively the only viable option, given the difficulty of sorting, and would avoid logistics-related emissions and costs [99]. The overall sustainability ranking will ultimately depend on facility capacity and the quality of the recovered fibers [100]. Solvents that preserve fiber quality well enough to replace virgin fibers 1:1 will outperform those that lead to downcycling, even if the latter achieve slightly higher recovery rates [100]. These variables can change the relative ranking of the routes. For example, a high-temperature route such as near-critical water hydrolysis could become competitive if process heat is supplied from renewable or recovered sources and the reactor is operated continuously, whereas a low-temperature route that relies on a large excess of solvent may lose its advantage if solvent recovery at scale is low. The positioning in Figure 14 and Table 9 should therefore not be read as a prediction of which route will necessarily prevail at industrial scale.

## 4. Conclusions and Outlook

This review systematically examined five principal chemical recycling pathways for anhydride-cured epoxy resins (hydrolysis, alcoholysis, glycolysis, aminolysis, and acetolysis), discussing the matrix degradation mechanisms and integrating a quantitative analysis of operational parameters and environmental hazard indicators. In almost all the studies compared, resin degradation exceeded 90% (range 84–100%) and frequently approached completion, confirming that anhydride-derived ester bonds are susceptible to cleavage by a broad range of chemical agents. Yet this convergence in conversion efficiency also clarifies where meaningful differentiation lies: not in whether a method works, but in the intensity of the process conditions and in the identity and volume of the solvents employed.

Hydrolysis in near-critical water avoids organic solvents and, in several studies, co-reactants, but requires high temperature and pressure, with the associated energy demand and process hazards. All the other approaches, including alcoholysis and glycolysis, can achieve full degradation at substantially lower temperatures, making solvent selection and volumes the dominant lever for environmental performance; on the other hand, the use of high-hazard co-solvents inflates SHE scores. Hence, solvent identity governs environmental hazard more strongly than process category. Moreover, none of the routes performs well against all the CHEM21 First Pass criteria: all are run in batch, most operate above 140 °C, and most use co-reactant loadings above 0.1 g per g of resin or CFRP.

Because degradation efficiency is uniformly high across methods, sustainable process development should be directed toward minimizing process intensity and solvent risk. Future work should therefore prioritize solvent recovery and closed-loop solvent management, in addition to techno-economic validation at industrial scale.

**Table 8:** Comparative overview of reaction conditions for selected solvolysis routes for anhydride-cured epoxy resins and CFRPs.

Reference	Process	Solvent	Co-Reactant or Catalyst	Solvent/Resin (or CFRP) (g/g) ^a^	Co-Reactant/Resin (or CFRP) (g/g) ^a^	Degrad. (%)	T (°C)	Time (min)
#Kim et al. (2019) [74]	Hydrolysis	Water (SCW) ^†^	n/a	n/r	n/a	99.5	405 ± 2 °C	120
Liu et al. (2012) [81] Gong et al. (2013) [82]	Hydrolysis	Water (NCW) ^†^	n/a	5.0	n/a	100	280–300	30
Backens et al. (2025) [84]	Hydrolysis	Water (NCW) ^†^	n/a	5.35	n/a	100	300	45
Liu et al. (2016) [76]	Alcoholysis	Methanol ^†^	KOH	7.9	0.02	100	210	120
Zhao et al. (2022) [85]	Alcoholysis	Ethanol ^†^	K_3_PO_4_	10.0	0.33	95.19 ± 3.68%	120	180
Zhang et al. (2025) [87]	Alcoholysis	Ethanol	K_3_PO_4_	10.0	0.33	≈100	120	180
Liu et al. (2025) [88]	Alcoholysis	Benzyl alcohol	K_3_PO_4_	5.0	0.50	100	190	60
#Ye et al. (2021) [77]	Alcoholysis	Benzyl alcohol	NaOH	8.4	0.84	>90	195	40
#Si et al. (2022) [71]	Alcoholysis	Benzyl alcohol	NaOH	62.7	1.00	98.4	190	60
#Min et al. (2025) [86]	Alcoholysis	β-Phenethyl alcohol	TBD	9.2	0.31	100	190	120
Zhang et al. (2018) [78]	Glycolysis	EG	N-methyl-4-piperidinol	7.7	0.15	99.7	180	180
#Tian et al. (2025) [90]	Glycolysis	EG	K_2_CO_3_	17.1	1.9	≈100	180	180
Zhao et al. (2023) [94]	Glycolysis	PEG 400 (DCM pre-treatment)	K_3_PO_4_	10.0	0.25	95	130	60
#Yang et al. (2014) [91]	Glycolysis	PEG 200	NaOH	20.0	0.10	84–93	200	240
#Kuang et al. (2018) [79]	Glycolysis	EG/NMP	TBD	11.2	0.45	95	170	90
Shi et al. (2022) [93]	Glycolysis	EG/NMP	TBD	n/r	n/r	100	160	34
#Mu et al. (2022) [69]	Glycolysis	DMF/EG	TBD	4.0	0.16	100	150	120
#Mu et al. (2022) [69]	Glycolysis	DMF/EG	Zn(acac)_2_	4.0	0.31	100	150	180
Zhao et al. (2023) [89]	Glycolysis	EG/DCM	NaOH	7.4	0.33	100	150	60
Zhao et al. (2019) [66]	Aminolysis	DETA (DCM pretreatment)	n/a	5.0	n/a	99	130	50
#Zhao et al. (2022) [70]	Aminolysis	MEA	KOH	9.1	0.20	99	163	60
Shao et al. (2023) [67]	Aminolysis	EA	n/a	4.0	n/a	100	160	240
#Yang et al. (2025) [95]	Aminolysis	EA	n/a	5.0	n/a	100	160	25
Lei et al. (2016) [68]	Acetolysis	Acetic anhydride	p-TSA	8.0	n/r	n/r	130	120

Cell colors for solvents and co-reactants indicate their ranking in Table 6 and Table 7 (green, recommended; yellow, problematic; red, hazardous); for solvent mixtures, the most hazardous component determines the color, and green is used where no co-reactant is added. ^a^ Mass ratios refer to the mass of resin or of CFRP, depending on the study, and are therefore not strictly comparable. All values are given as reported in the original studies; missing values are marked n/r. All experiments were carried out at laboratory scale in batch. Studies on CFRP are flagged with #; ^†^ Process operating above atmospheric pressure. n/r = not reported; SCW = supercritical water; NCW = near-critical water; EG = ethylene glycol; PEG = poly(ethylene glycol); NMP = N-methyl-2-pyrrolidone; DMF = N,N-dimethylformamide; DCM = dichloromethane; DETA = diethylenetriamine; EA/MEA = ethanolamine; TBD = 1,5,7-triazabicyclo [4.4.0]dec-5-ene; Zn(acac)_2_ = zinc acetylacetonate; p-TSA = p-toluenesulfonic acid.

**Table 9:** CHEM21 First Pass flags for the solvolysis studies listed in Table 8.

Reference	Process	Catalyst/Co-Reactant Use	Critical Elements	Energy (T)	Reflux (T_eb_)	Batch/Flow	Chemicals of Concern
Kim et al. (2019) [74]	Hydrolysis †	n/a	G	R (405 °C)	n/a	A	G
Liu et al. (2012) [81]	Hydrolysis †	n/a	G	R (280–300 °C)	n/a	A	G
Backens et al. (2025) [84]	Hydrolysis †	n/a	G	R (300 °C)	n/a	A	G
Liu et al. (2016) [76]	Alcoholysis †	G (0.02)	G	R (210 °C)	n/a	A	G
Zhao et al. (2022) [85]	Alcoholysis †	A (0.33)	A (P)	Y (120 °C)	n/a	A	G
Zhang et al. (2025) [87]	Alcoholysis	A (0.33)	A (P)	Y (120 °C)	n/a	A	G
Liu et al. (2025) [88]	Alcoholysis	A (0.50)	A (P)	R (190 °C)	G (205 °C)	A	G
Ye et al. (2021) [77]	Alcoholysis	A (0.84)	G	R (195 °C)	G (205 °C)	A	G
Si et al. (2022) [71]	Alcoholysis	A (1.00)	G	R (190 °C)	G (205 °C)	A	G
Min et al. (2025) [86]	Alcoholysis	A (0.31)	G	R (190 °C)	G (219 °C)	A	G
Zhang et al. (2018) [78]	Glycolysis	A (0.15)	G	R (180 °C)	G (197 °C)	A	G
Tian et al. (2025) [90]	Glycolysis	A (1.9)	G	R (180 °C)	G (197 °C)	A	G
Zhao et al. (2023) [94]	Glycolysis	A (0.25)	A (P)	Y (130 °C)	G (PEG 400)	A	A (DCM)
Yang et al. (2014) [91]	Glycolysis	G (0.10)	G	R (200 °C)	G (PEG 200)	A	G
Kuang et al. (2018) [79]	Glycolysis	A (0.45)	G	R (170 °C)	G	A	R (NMP)
Shi et al. (2022) [93]	Glycolysis	n/r	G	R (160 °C)	G	A	R (NMP)
Mu et al. (2022) [69] (TBD)	Glycolysis	A (0.16)	G	R (150 °C)	R (153 °C)	A	R (DMF)
Mu et al. (2022) [69] (Zn(acac)_2_)	Glycolysis	A (0.31)	G	R (150 °C)	R (153 °C)	A	R (DMF)
Zhao et al. (2023) [89]	Glycolysis	A (0.33)	G	R (150 °C)	G (197 °C)	A	A (DCM)
Zhao et al. (2019) [66]	Aminolysis	n/a	G	Y (130 °C)	G (207 °C)	A	A (DCM)
Zhao et al. (2022) [70]	Aminolysis	A (0.20)	G	R (163 °C)	G (170 °C)	A	G
Shao et al. (2023) [67]	Aminolysis	n/a	G	R (160 °C)	G (170 °C)	A	G
Yang et al. (2025) [95]	Aminolysis	n/a	G	R (160 °C)	G (170 °C)	A	G
Lei et al. (2016) [68]	Acetolysis	n/r	G	Y (130 °C)	G (139 °C)	A	G

G = green; A = amber; Y = yellow; R = red; n/a = not applicable (no catalyst/co-reactant, or process run under pressure); n/r = catalyst/co-raectant amount is not reported n/r; Catalyst/co-reactantuse: loading (g per g of resin or CFRP) in brackets; green ≤ 0.1 g/g, amber > 0.1 g/g. Energy: reaction temperature in brackets. Reflux: atmospheric boiling point of the solvent in brackets; not applicable to processes run under pressure. Chemicals of concern: substances restricted under REACH or listed as Substances of Very High Concern. P = phosphorus (EU critical raw material); NMP = N-methyl-2-pyrrolidone; DMF = N,N-dimethylformamide; DCM = dichloromethane. † Process operating above atmospheric pressure.

**Table 10:** Issues and considerations for the scale-up of the different chemical recycling processes.

Solvolysis Route	Scale-Up Issue	Main Technical Issues	Sustainability Considerations	Source
Hydrolysis	High T/p --> reactor capex and energy demand	Batch to continuous, process heat	High energy demand can offset recycling benefit; heat and energy supply needs to be low-carbon	[101,106,107]
Alcoholysis	Choice of alcohol and recovery under pressure; flammability	Batch to continuous, solvent recovery, process heat	Production and recovery of solvent influence LCA strongly;	[102]
Glycolysis	Catalyst/solvent recovery and reaction time	Process heat	Strongly dependent on glycol type, catalyst and reaction time	[91]
Aminolysis	Chemical recovery/separation	Amine recovery and pretreatment	Amine consumption and recovery; chemical toxicity and corrosivity	[66]
Acetolysis	Most developed route at the moment	Acid recovery, purification	Sustainability of the process depends strongly on acid recovery	[104,105]

A final consideration concerns the inherent limitations of solvolysis when applied to highly heterogeneous composite waste streams. Unlike mechanical recycling, which can process mixed feedstocks with relative tolerance, solvolysis is strongly dependent on the chemical nature of the matrix: reaction conditions optimized for one resin system may be ineffective when applied to a mixture of different thermoset chemistries. Process efficiency, fiber quality, and solvent recovery are all compromised when the feedstock is poorly sorted. Consequently, solvolysis is best suited to treating single-matrix composite waste, where reaction parameters can be precisely tuned to maximize both fiber reclamation yield and solvent recyclability, while minimizing energy consumption and chemical waste. This constraint has an important practical implication: rather than centralizing composite waste treatment at large, generalist recycling facilities, a promising strategy, still to be confirmed by quantitative assessment, may be to deploy solvolysis at or near the sites where composite materials are produced and used. Aerospace plants, wind turbine manufacturers, and automotive production lines generate relatively homogeneous off-cuts, end-of-life tooling, and end-of-service components whose matrix chemistry is well-characterized. Integrating solvolysis, and ideally the downstream remanufacturing of reclaimed fibers, directly into these industrial ecosystems would close the material loop at source, reduce logistics-related emissions, and ensure the feedstock consistency that the process demands.

Because most solvolysis routes have been studied only at laboratory scale, their environmental performance at scale remains uncertain; it will depend on low-temperature operation powered by renewable energy, a shift from batch to continuous processing, on-site deployment, facility capacity, and, above all, recovered-fiber quality.

The challenge for the scientific and engineering community, therefore, is two-fold: to develop solvolysis protocols that are robust, scalable, and economically viable at the industrial-site level, and to couple them with fiber reprocessing routes capable of delivering reclaimed reinforcements that meet the quality standards required for re-entry into structural or semi-structural applications. Achieving this would represent a significant step toward genuine circularity in composite manufacturing.

## Figures and Tables

**Figure 1:**
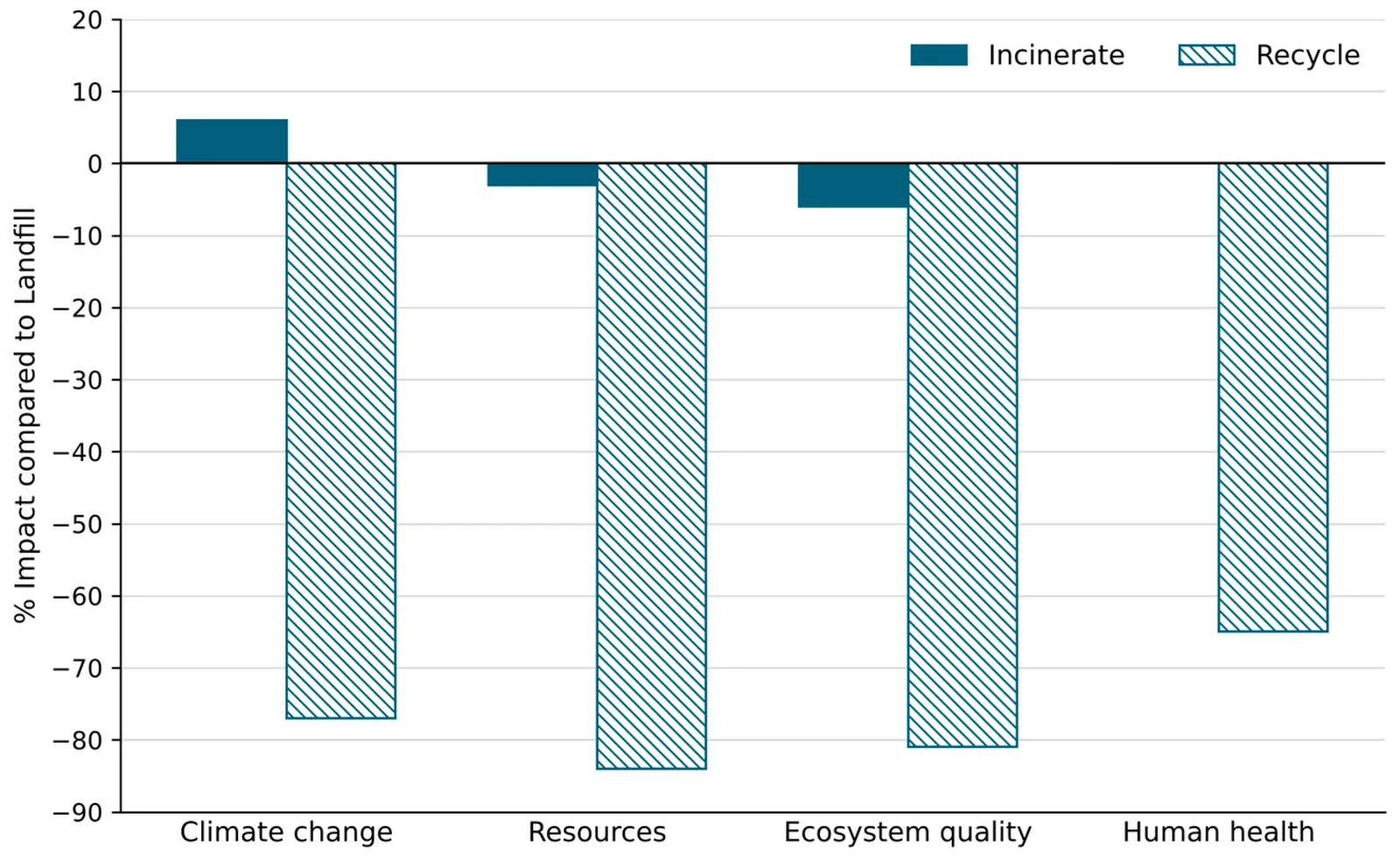
Environmental impact of incineration with energy recovery and recycling of carbon fiber-reinforced composites, expressed as percentage change relative to landfill disposal (0% = landfill; negative values indicate a lower impact than landfill). Data from ref. [13].

**Figure 2:**
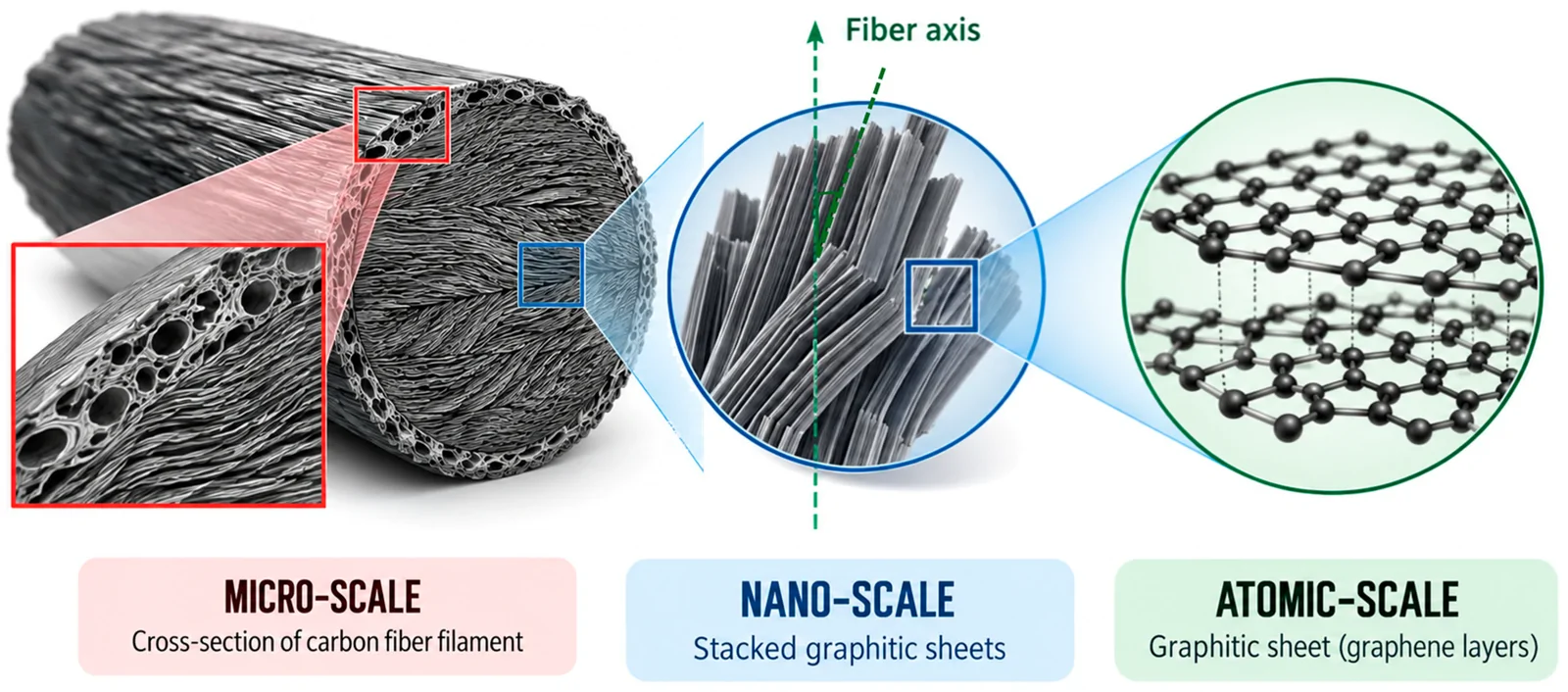
Carbon fiber microstructure across atomic, nano, and microscales.

**Figure 3:**
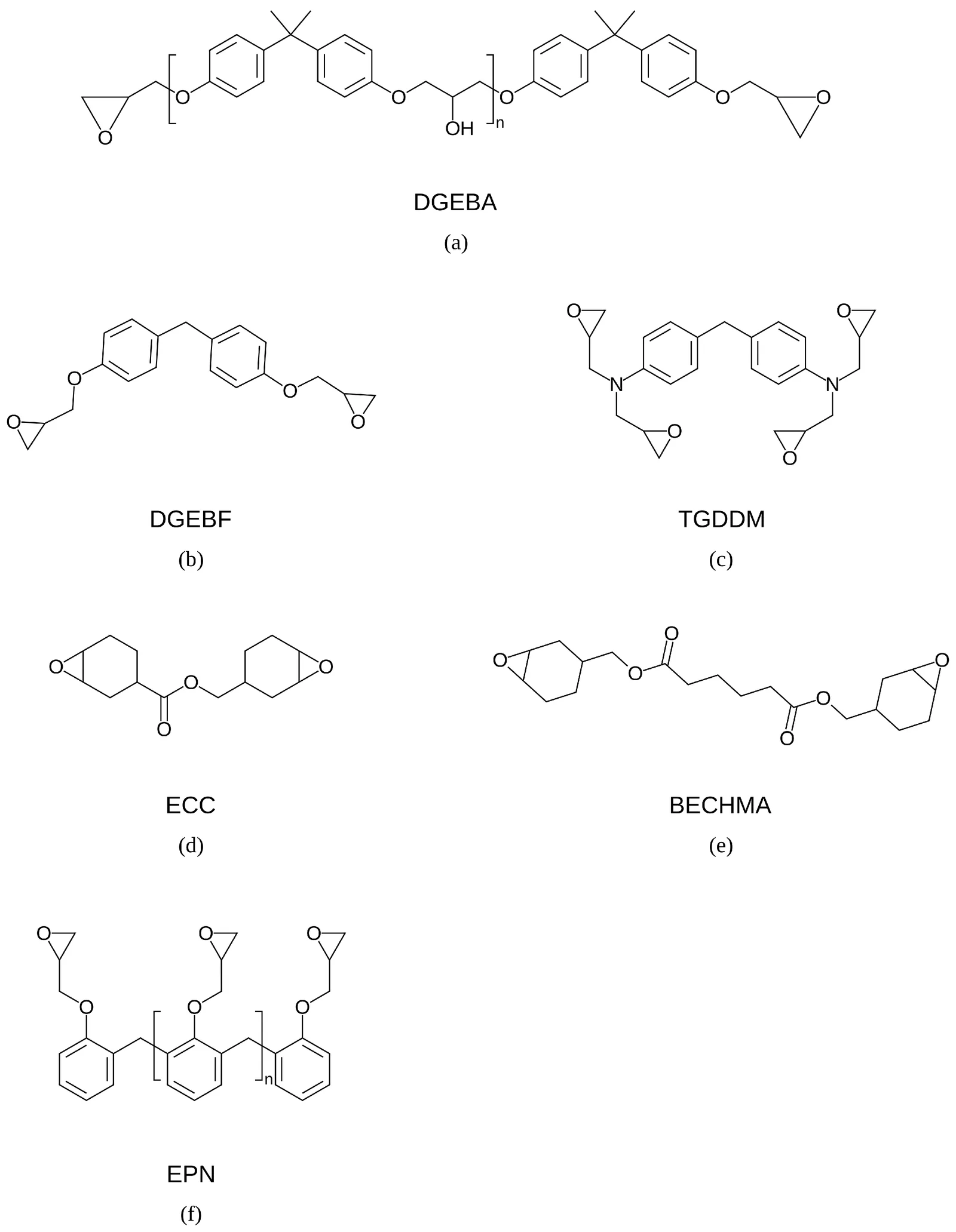
Chemical structures of common epoxy resins: (**a**) diglycidyl ether of bisphenol A (DGEBA); (**b**) diglycidyl ether of bisphenol F (DGEBF); (**c**) N,N,N′,N′-tetraglycidyl-4,4′-diaminodiphenylmethane (TGDDM); (**d**) 3,4-epoxycyclohexylmethyl 3,4-epoxycyclohexanecarboxylate (ECC); (**e**) bis(3,4-epoxycyclohexylmethyl) adipate (BECHMA); (**f**) epoxy phenol novolac (EPN). In (**a**,**f**), *n* is the number of repeat units. In (**f**), ortho-linked rings are shown as a representative structure; commercial EPN contains a mixture of o,o′-, o,p′- and p,p′-methylene linkages.

**Figure 4:**
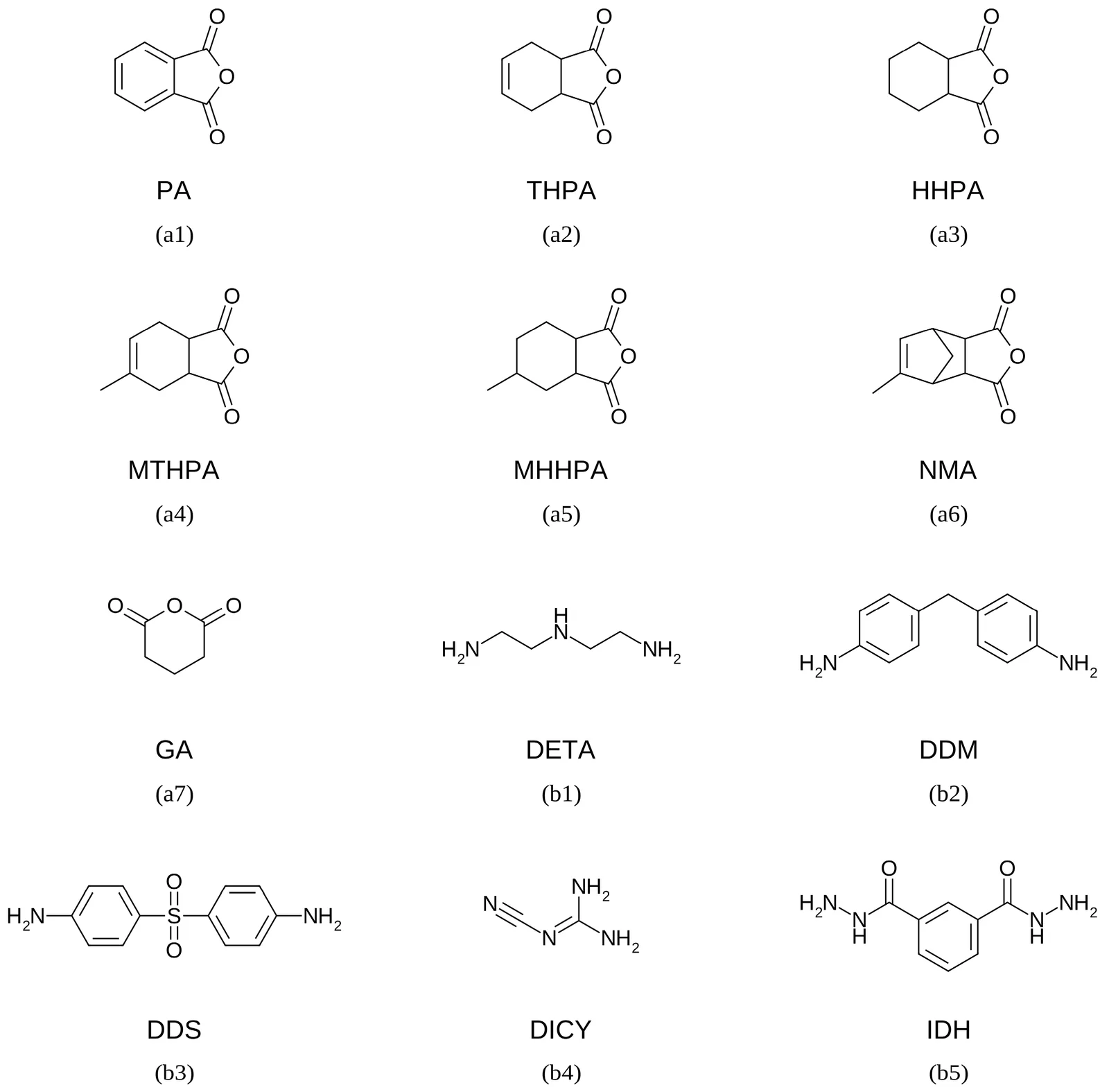
Chemical structures of common epoxy curing agents. Anhydride hardeners: (**a1**) phthalic anhydride (PA); (**a2**) cis-1,2,3,6-tetrahydrophthalic anhydride (THPA); (**a3**) hexahydrophthalic anhydride (HHPA or HPA); (**a4**) methyl tetrahydrophthalic anhydride (MTHPA); (**a5**) methyl hexahydrophthalic anhydride (MHHPA); (**a6**) nadic methyl anhydride (NMA, methyl-5-norbornene-2,3-dicarboxylic anhydride); (**a7**) glutaric anhydride (GA). Nitrogen-based hardeners: (**b1**) diethylenetriamine (DETA); (**b2**) 4,4′-diaminodiphenylmethane (DDM); (**b3**) 4,4′-diaminodiphenyl sulfone (DDS); (**b4**) dicyandiamide (DICY, 2-cyanoguanidine); (**b5**) isophthalic dihydrazide (IDH). MTHPA, MHHPA and NMA are commercial mixtures of isomers, and one representative isomer of each is shown; stereochemistry is not shown.

**Figure 5:**
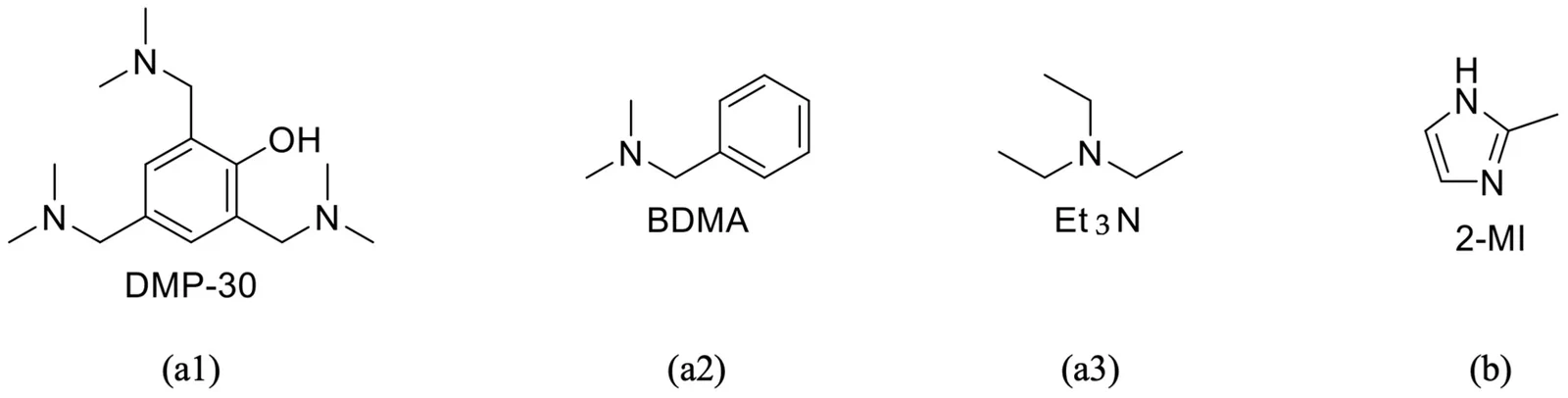
Chemical structures of common epoxy-anhydride accelerators: (**a1**) 2,4,6-tris(dimethylaminomethyl)phenol (DMP-30), (**a2**) benzyldimethylamine (BDMA) and (**a3**) triethylamine (Et3N), (**b**) 2-methylimidazole (2-MI).

**Figure 6:**
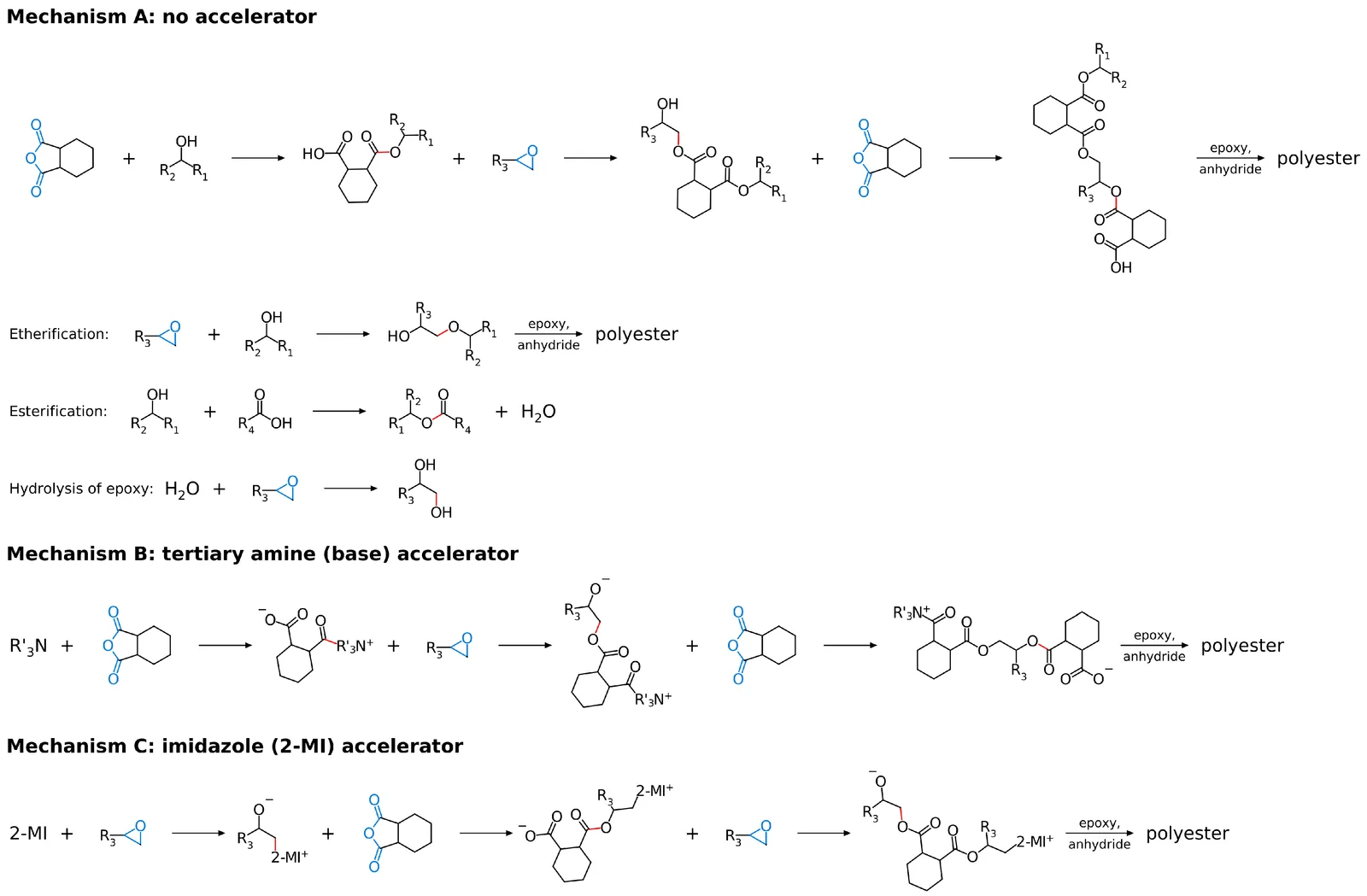
Examples of epoxy–anhydride curing processes that proceed via anhydride ring opening by hydroxyl groups, forming β-hydroxy esters and driving polyester network growth, with competing side reactions including etherification and epoxy hydrolysis (mechanism A). Tertiary amine accelerators activate the anhydride, generating carboxylate anions that react alternately with epoxide groups in an autocatalytic, anhydride-activated mechanism (mechanism B). Imidazole accelerators instead initiate curing by nucleophilic epoxide opening, with the resulting alkoxide driving an epoxide-activated propagation pathway through alternating ester bond formation and carboxylate regeneration (mechanism C). Epoxy and anhydride functional groups are shown in blue. The bonds formed in the curing reactions are shown in red. Mechanisms according to ref [35].

**Figure 7:**
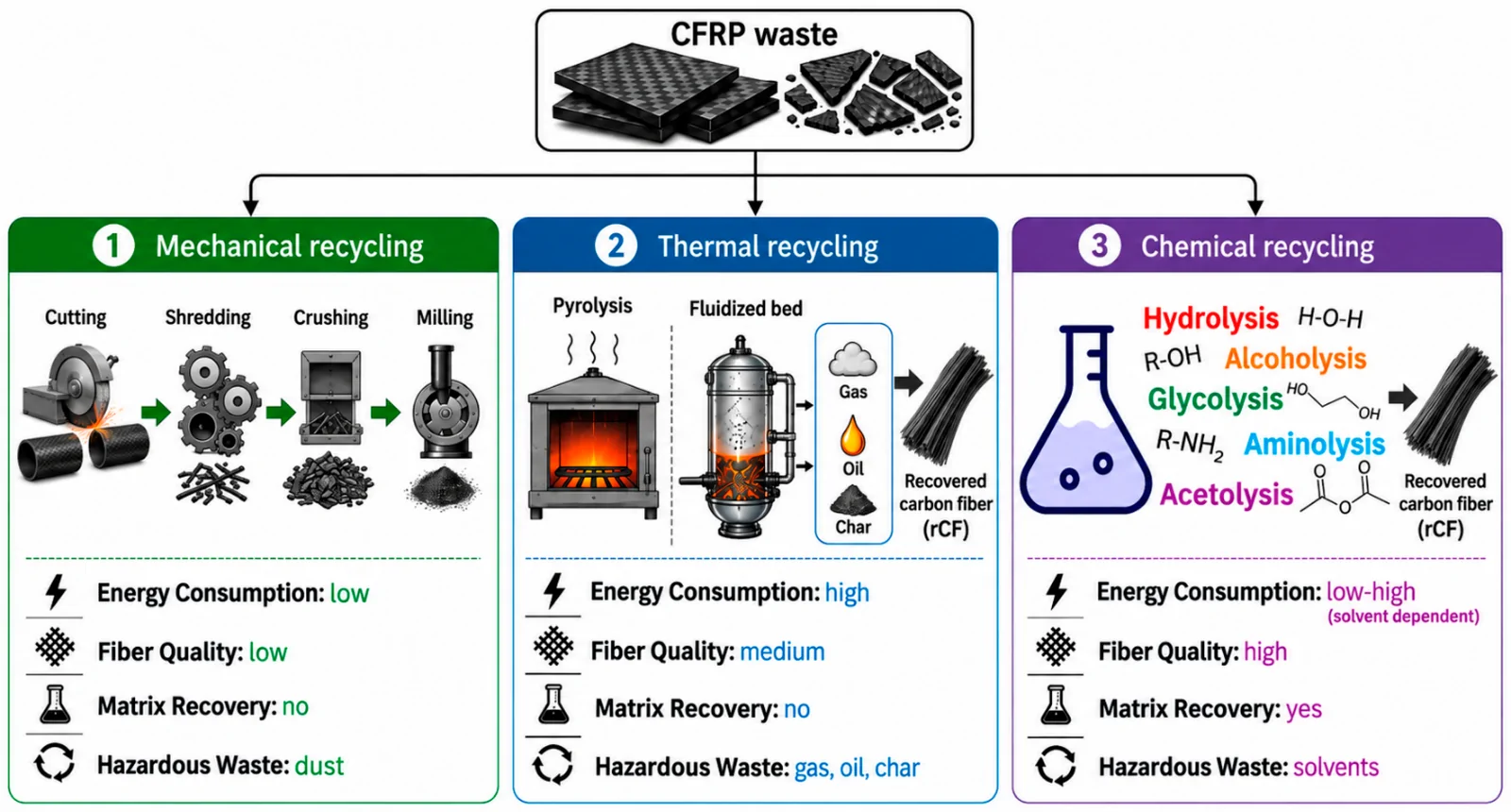
Schematic overview of the main recycling routes for CFRP waste.

**Figure 8:**
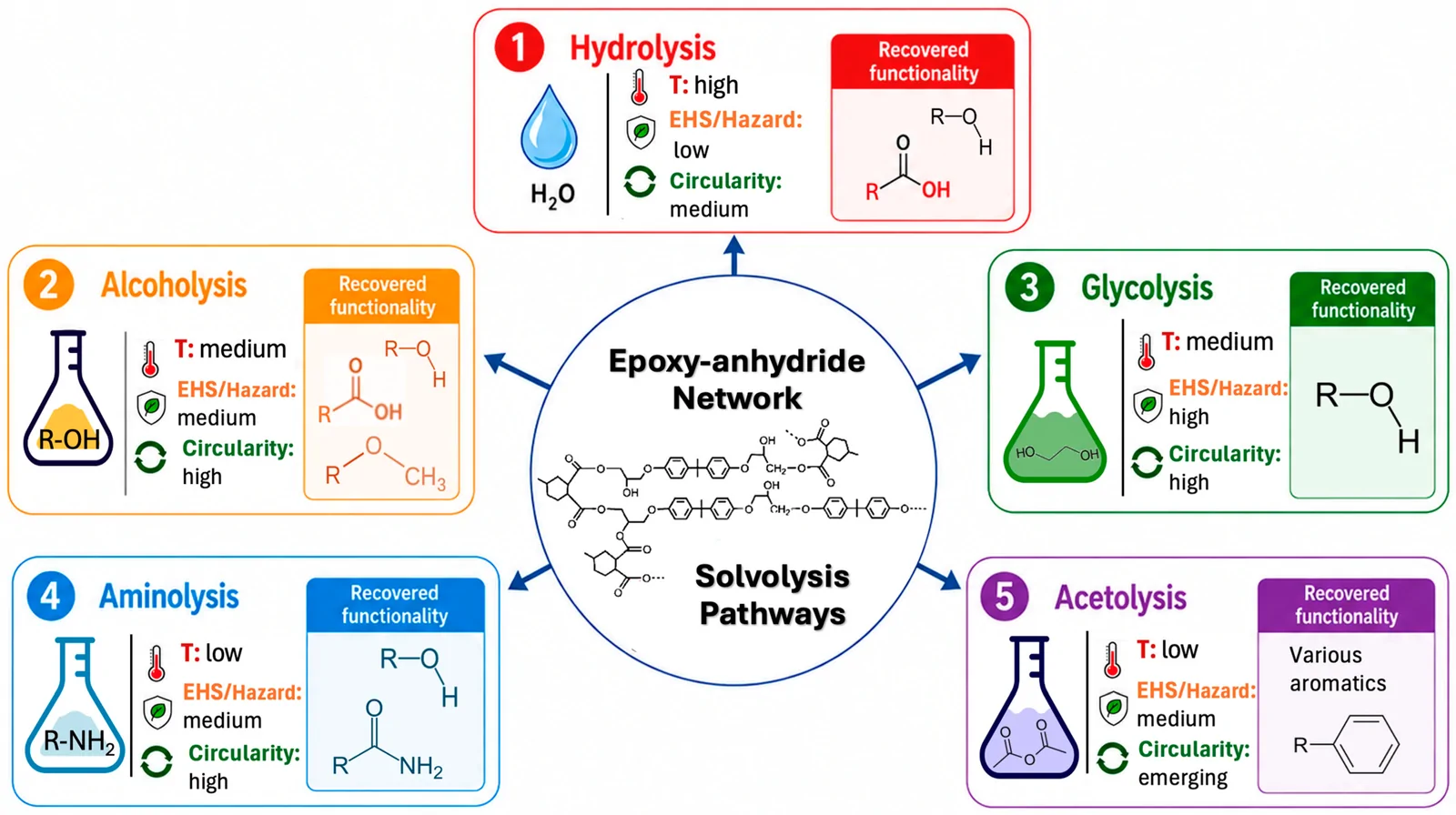
Schematic comparison of the main solvolysis pathways for anhydride-cured epoxy networks. Hydrolysis, alcoholysis, glycolysis, aminolysis, and acetolysis are compared in terms of typical operating temperature, SHE/hazard profile, circularity potential based on the possibility of solvent recovery, and the main functionalities recovered after network cleavage.

**Figure 9:**
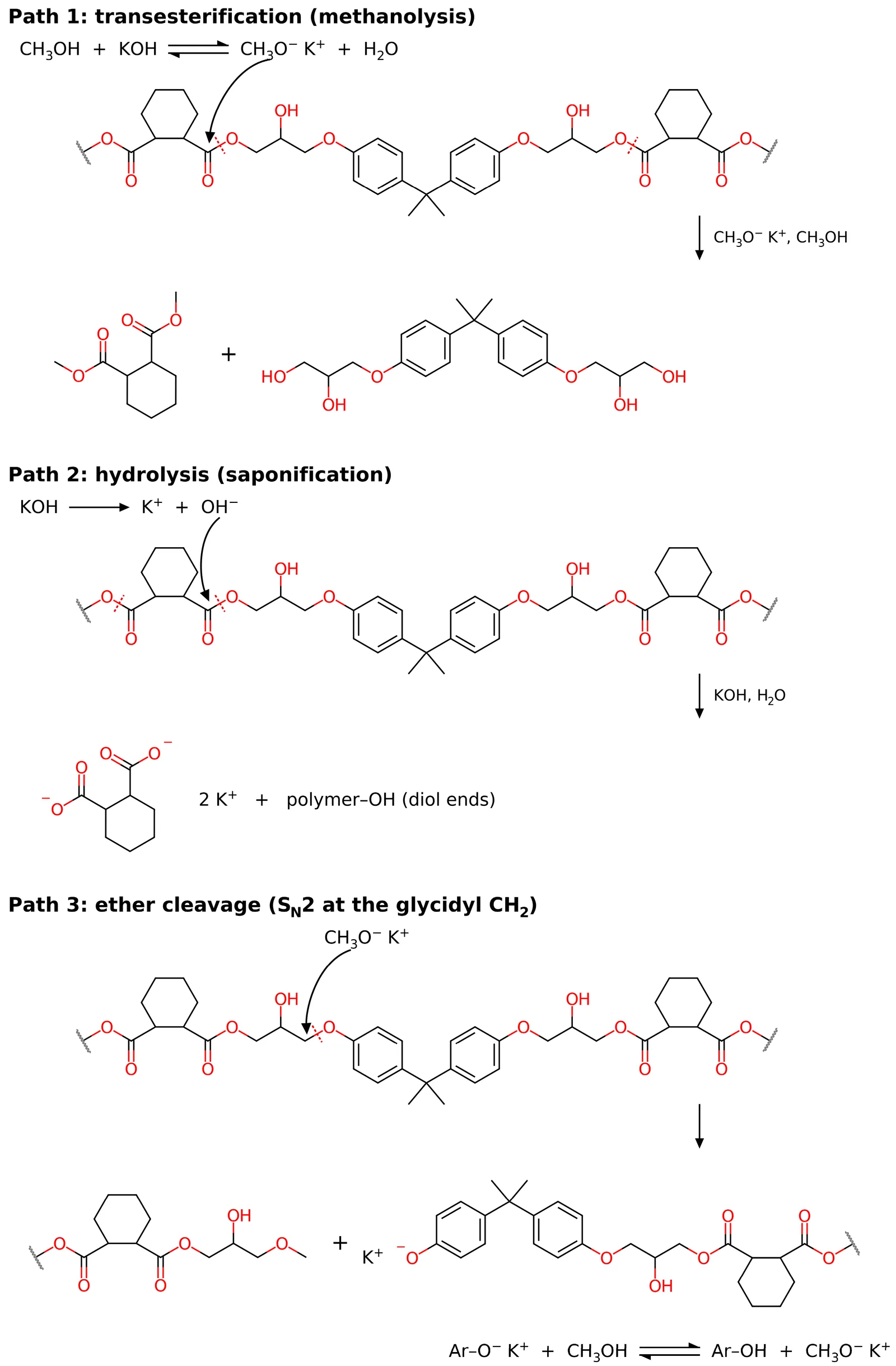
Example of the mechanism of degradation of epoxy resin cured with hexahydrophthalic anhydride in subcritical methanol in the presence of KOH. Curved arrows indicate nucleophilic attack: by methoxide on the ester carbonyl carbon (Path 1), by hydroxide on the ester carbonyl carbon (Path 2), and by methoxide on the glycidyl CH_2_ carbon adjacent to the aryl ether oxygen (Path 3). Red dashed lines mark the bonds cleaved in each pathway. Oxygen atoms in ethers and esters, hydroxyl groups and the bonds to them are shown in red; wavy lines indicate continuation of the polymer network. Mechanism according to Ref. [76].

**Figure 10:**
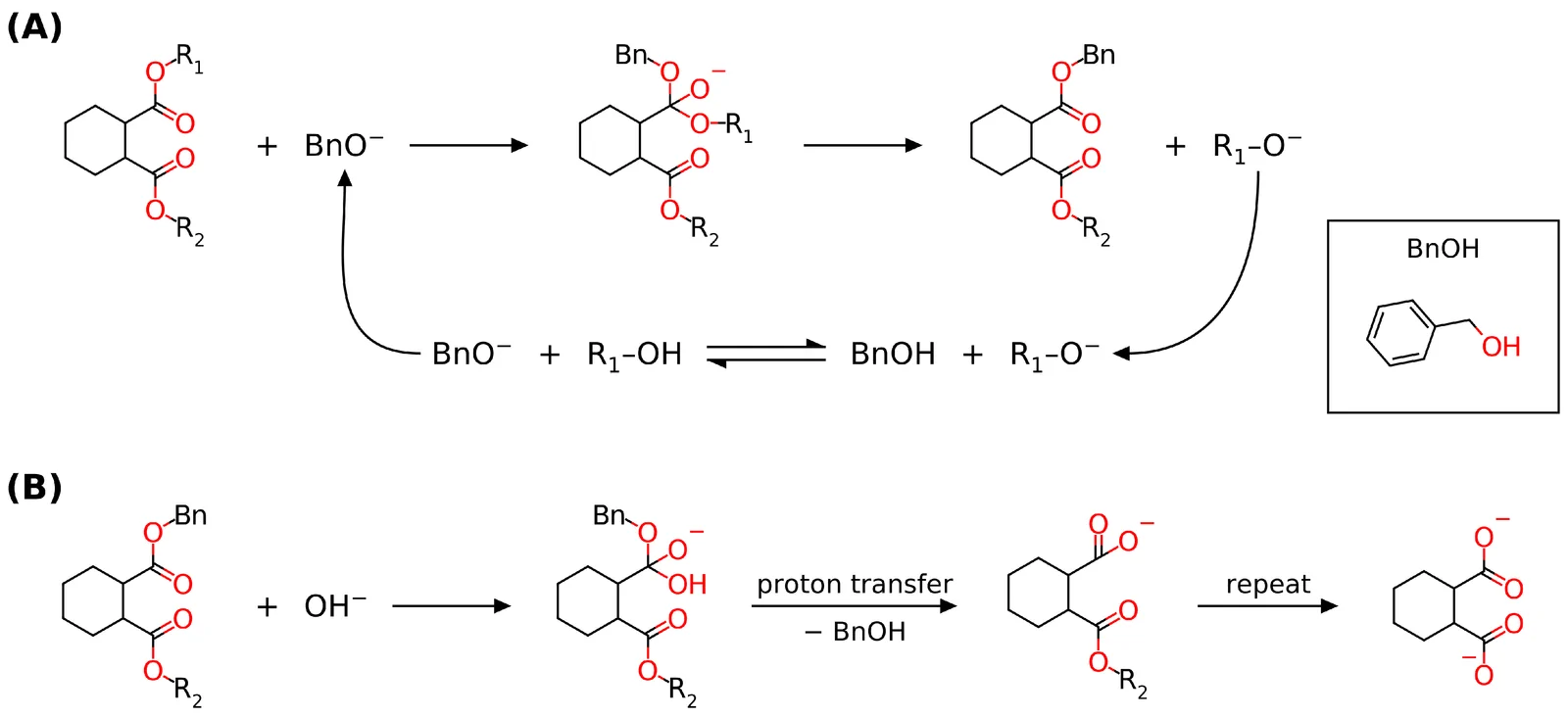
Example of transesterification (**A**) and saponification (**B**) of epoxy–anhydride resin cleavage by benzyl alcohol/K_3_PO_4_ or benzyl alcohol/NaOH. Benzylate (BnO^−^), formed by deprotonation of benzyl alcohol by the base, attacks the ester carbonyl to give a tetrahedral intermediate that transforms into a benzyl ester, releasing the network alkoxide R_1_–O^−^. Proton exchange between R_1_–O^−^ and benzyl alcohol releases the hydroxyl-terminated network fragment (R_1_–OH) and regenerates BnO^−^, so the base acts catalytically. Oxygen atoms in ethers and esters, hydroxyl groups and the bonds to them are shown in red. Mechanism according to Ref. [77].

**Figure 11:**
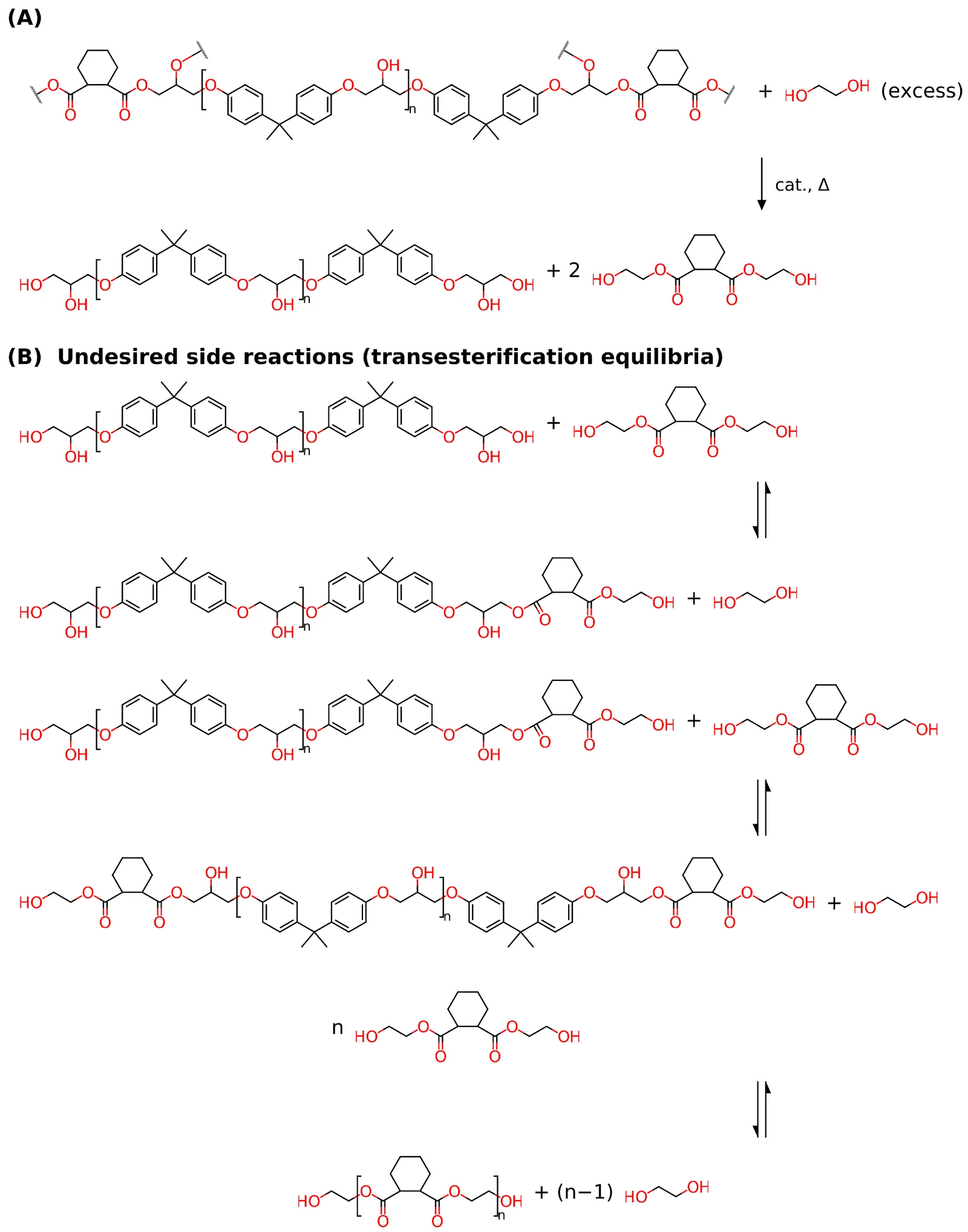
(**A**) Curing of DGEBA with HP forming a crosslinked polyester-like epoxy–anhydride network through ester linkages. (**B**) Transesterification solvolysis of the cured network in diethylene glycol, yielding tetraalcohol and dihydroxyalkyl ester fragments, with possible secondary exchange reactions. Oxygen atoms in ethers and esters, hydroxyl groups and the bonds to them are shown in red. Mechanism according to Ref. [92].

**Figure 12:**
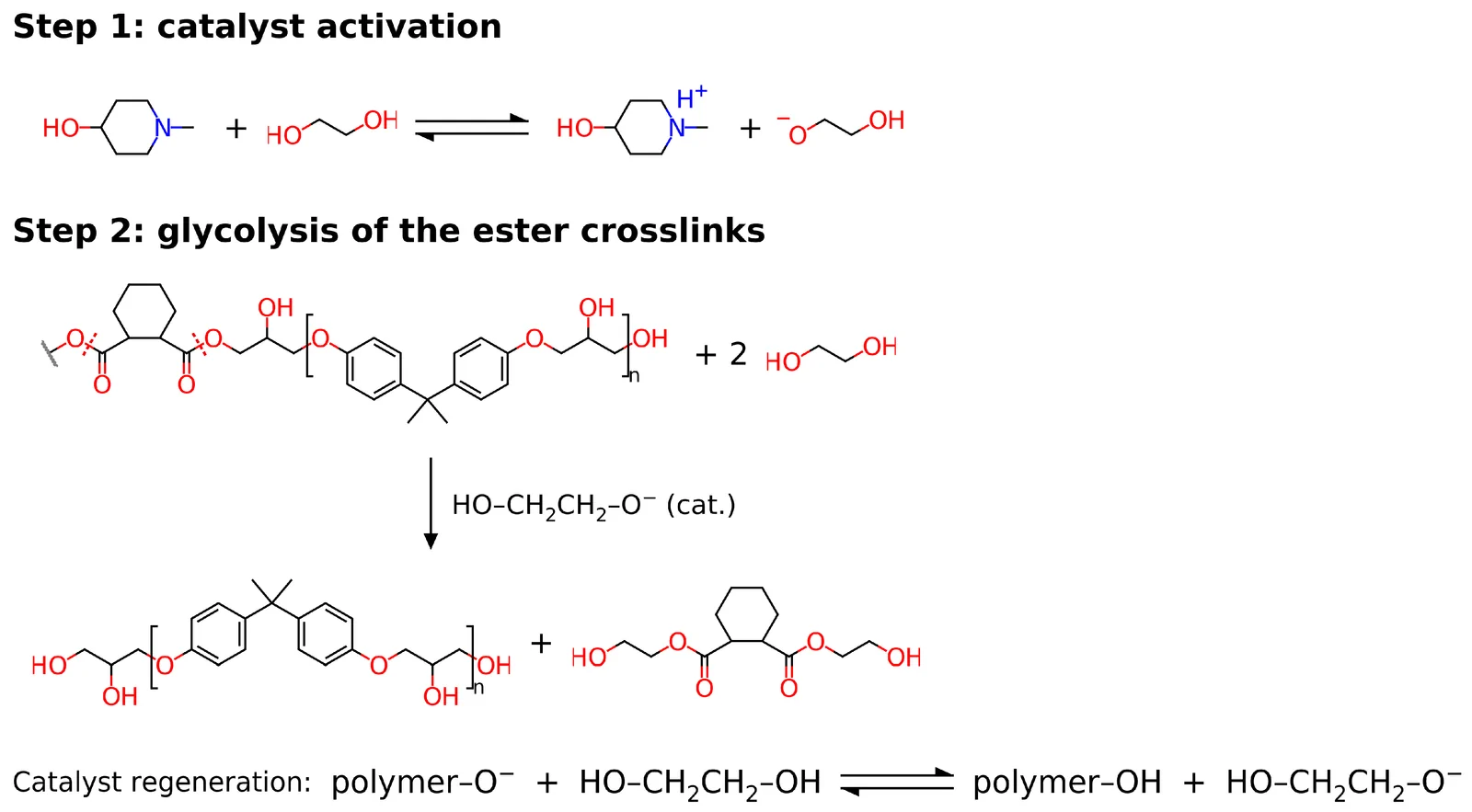
Mechanism of the degradation of an an epoxy–anhydride resin by ethylene glycol in the presence of N-methyl-4-piperidinol as organic base catalyst. Step 1: the tertiary amine partially deprotonates the glycol, generating the 2-hydroxyethoxide anion and the protonated piperidinium cation. Step 2: the 2-hydroxyethoxide attacks the carbonyl carbon of the anhydride-derived ester linkages; red dashed lines mark the ester bonds that are cleaved. Transesterification releases the bisphenol A-based segments as hydroxyl-terminated oligomers (diol/tetraol ends) and converts the anhydride residues into bis(2-hydroxyethyl) hexahydrophthalate. Catalyst regeneration: the polymer alkoxide formed after cleavage is protonated by EG, restoring the 2-hydroxyethoxide, so the base acts catalytically. The anhydride unit is represented as hexahydrophthalate. The wavy lines indicate continuation of the network. Oxygen atoms in ethers and esters and hydroxyl groups are shown in red and nitrogen atoms in blue. Mechanism according to [78].

**Figure 13:**
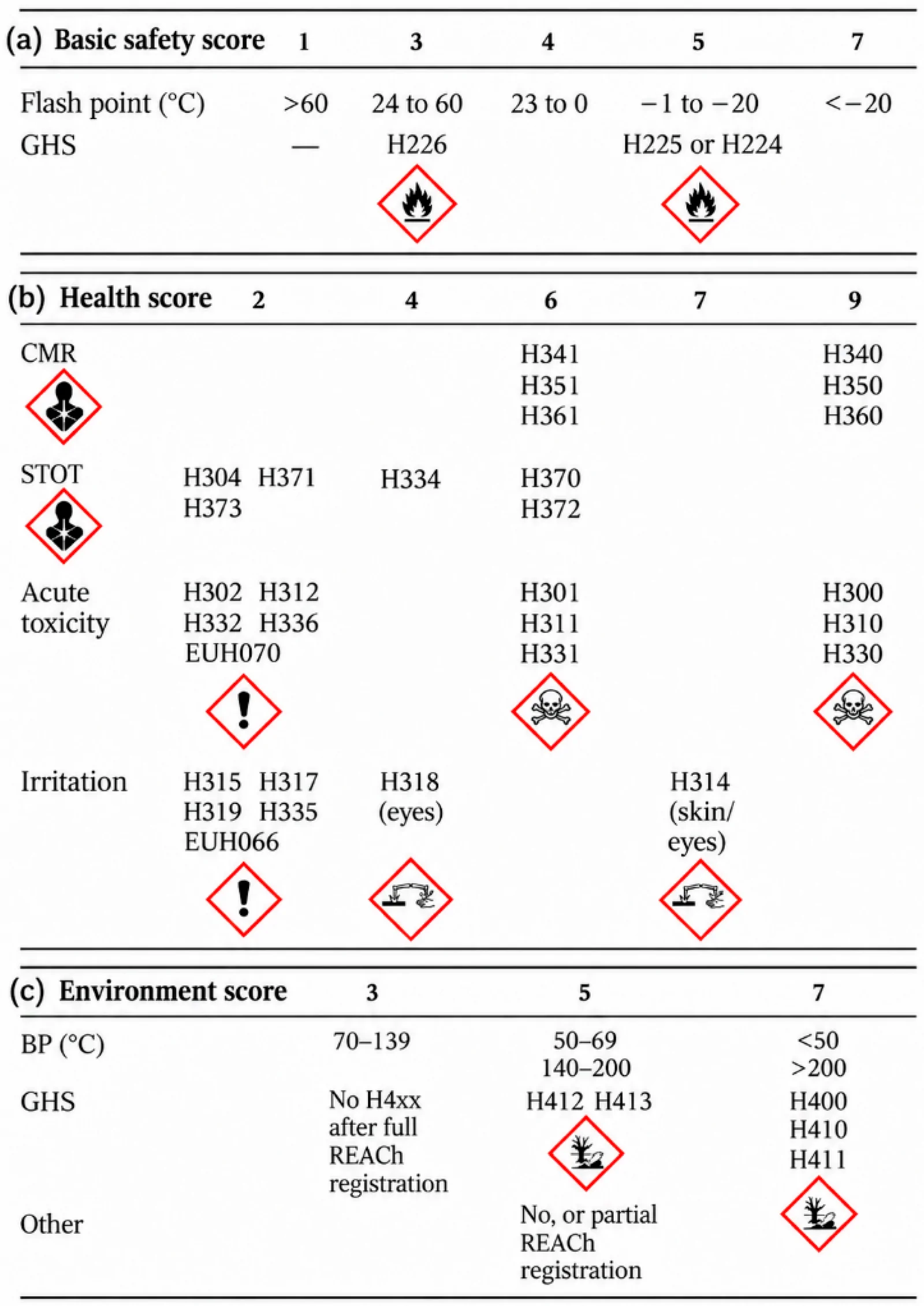
SHE scoring criteria for solvent evaluation. (**a**) Safety scoring system based on flash point, GHS flammability classification, and additional fire-related hazards. (**b**) Health scoring system derived from GHS hazard statements, including CMR, STOT, acute toxicity, and irritation categories. (**c**) Environmental scoring system based on boiling point range, aquatic toxicity (H4xx), ozone hazard, and REACh registration status [reproduced from Ref. [97] under the terms of the Creative Commons CC BY-NC 3.0 license].

**Figure 14:**
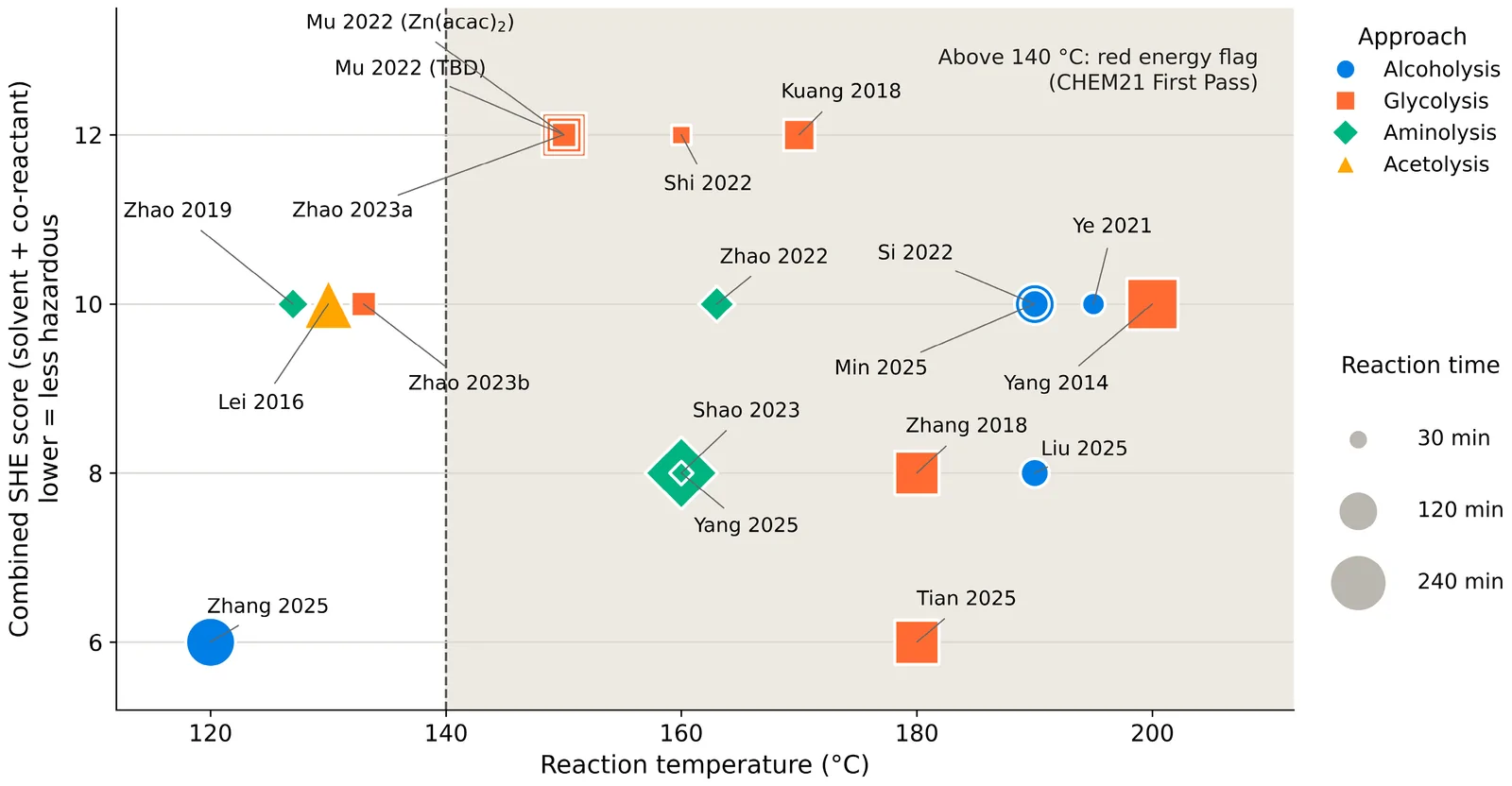
SHE hazard screening versus reaction temperature for solvolysis approaches to anhydride-cured epoxy resins and composites operating at atmospheric pressure. The combined SHE score is the sum of the solvent and co-reactant category scores (recommended = 3, problematic = 5, hazardous = 7; 3 when no co-reactant is used; for solvent mixtures, the score of the most hazardous component). Marker shape and colour identify the solvolysis approach; marker area is proportional to reaction time, with equal area for equal time irrespective of shape (see the reaction-time legend). Studies with identical temperature and score are shown as concentric markers; where such studies belong to different approaches (Zhao 2019, Lei 2016 and Zhao 2023b, all at 130 °C), the markers are offset horizontally by 3 °C for legibility. The shaded area marks temperatures above 140 °C, which receive a red energy flag in the CHEM21 First Pass. Processes operating under pressure are not shown (see Table 8). Data from: Zhang 2025 [87], Si 2022 [71], Ye 2021 [77], Min 2025 [86], Liu 2025 [88], Zhang 2018 [78], Tian 2025 [90], Zhao 2023a [89], Zhao 2023b [94], Yang 2014 [91], Kuang 2018 [79], Shi 2022 [93], Mu 2022 [69], Zhao 2019 [66], Zhao 2022 [70], Shao 2023 [67], Yang 2025 [95], Lei 2016 [68].

## Data Availability

No new data were created or analyzed in this study. Data sharing is not applicable to this article.
